# A single amino acid polymorphism in natural Metchnikowin alleles of *Drosophila* results in systemic immunity and life history tradeoffs

**DOI:** 10.1371/journal.pgen.1011155

**Published:** 2024-03-11

**Authors:** Jessamyn I. Perlmutter, Joanne R. Chapman, Mason C. Wilkinson, Isaac Nevarez-Saenz, Robert L. Unckless

**Affiliations:** 1 Department of Molecular Biosciences, University of Kansas, Lawrence, Kansas, United States of America; 2 Institute of Environmental and Scientific Research (ESR), Christchurch, New Zealand; 3 Department of Biochemistry, Vanderbilt University, Nashville, Tennessee, United States of America; Univ. Maryland Baltimore County, UNITED STATES

## Abstract

Antimicrobial peptides (AMPs) are at the interface of interactions between hosts and microbes and are therefore expected to be rapidly evolving in a coevolutionary arms race with pathogens. In contrast, previous work demonstrated that insect AMPs tend to evolve more slowly than the genome average. Metchikowin (Mtk) is a *Drosophila* AMP that has a single amino acid residue that segregates as either proline (P) or arginine (R) in populations of four different species, some of which diverged more than 10 million years ago. These results suggest that there is a distinct functional importance to each allele. The most likely hypotheses are driven by two main questions: does each allele have a different efficacy against different specific pathogens (specificity hypothesis)? Or, is one allele a more potent antimicrobial, but with a host fitness cost (autoimmune hypothesis)? To assess their functional differences, we created *D*. *melanogaster* lines with the P allele, R allele, or *Mtk* null mutation using CRISPR/Cas9 genome editing and performed a series of life history and infection assays to assess them. In males, testing of systemic immune responses to a repertoire of bacteria and fungi demonstrated that the R allele performs as well or better than the P and null alleles with most infections. Females show some results that contrast with males, with *Mtk* alleles either not contributing to survival or with the P allele outperforming the R allele. In addition, measurements of life history traits demonstrate that the R allele is more costly in the absence of infection for both sexes. These results are consistent with both the specificity hypothesis (either allele can perform better against certain pathogens depending on context), and the autoimmune hypothesis (the R allele is generally the more potent antimicrobial in males, and carries a fitness cost). These results provide strong *in vivo* evidence that differential fitness with or without infection and sex-based functional differences in alleles may be adaptive mechanisms of maintaining immune gene polymorphisms in contrast with expectations of rapid evolution. Therefore, a complex interplay of forces including pathogen species and host sex may lead to balancing selection for immune genotypes. Strikingly, this selection may act on even a single amino acid polymorphism in an AMP.

## Author summary

Antimicrobial peptides (AMPs) are small peptides produced by many living organisms as a frontline immune response to a variety of microbial pathogens. These peptides directly interface with pathogens to kill them shortly after infection. Importantly, multiple AMP alleles can be balanced within a population, but little is known about how this immune variation among individuals is maintained over time. Multiple species of *Drosophila* balance two specific alleles of the AMP, Metchnikowin (Mtk), a canonically antifungal and antibacterial peptide. Here, various life-history and infection assays assess the mechanism of Metchnikowin balancing selection using CRISPR-generated *D*. *melanogaster* with a null mutation or either of the two alleles, *Mtk*^*R*^ or *Mtk*^*P*^. Findings demonstrate that the *Mtk*^*R*^ allele usually confers greater protection than the *Mtk*^*P*^ allele in males, but carries a fitness cost without infection. However, this pattern flips in females with certain infections, with the *Mtk*^*P*^ allele conferring greater protection than *Mtk*^*R*^. This suggests that the two alleles are repeatedly maintained in fly populations due to differential fitness of hosts in the context of many variables including host genotype, sex, infection status, and pathogen species. It also suggests that maintenance of immune variation in a population can be based on just a single amino acid difference between individuals.

## Introduction

Animals must maintain an intricate balance between their immune reactions to pathogens and other traits associated with host fitness and health [1–5]. This is due to a necessary tradeoff between protecting from numerous encounters with harmful microbes while also avoiding detrimental side effects of autoimmune reactions [5–8]. Antimicrobial peptides are at the forefront of animal immune defenses, particularly for invertebrates that rely solely on an innate immune system [9–11]. Invertebrate innate immune defenses against bacteria and fungi are primarily regulated by the immune-deficiency pathway (Imd) (response mainly to Gram-negative, some Gram-positive bacteria) [12] and the Toll pathway (response mainly to Gram-positive bacteria and fungi) [11,13,14]. When harmful microbes are sensed through various pathogen-associated molecular patterns such as the peptidoglycan in bacterial cell walls, a signaling cascade through the Imd and Toll receptors of host cells is initiated [15]. This leads to downstream transcription and translation of molecules including antimicrobial peptides (AMPs) [16,17]. AMPs are generally short, cationic peptides (about 12–50 amino acids) that are often post-translationally processed and secreted from host cells [17,18]. These peptides directly interact with pathogens and kill or impede them through mechanisms such as destabilization of membranes or inhibition of essential processes like translation [19,20]. However, AMPs and other immune genes are regulated temporally and spatially to avoid harm to the host, as expression of immune molecules can be detrimental due to excessive inflammation, off-target effects, the energetic cost of AMP expression, or other factors [5,21–26]. For example, the Diptericin AMP in *Drosophila* is one case of a peptide that likely carries a fitness cost as evidenced by recurrent loss and pseudogenization in Tephritids and Drosophilids [27].

Although population-level immune gene variation can be critical for keeping up with an ever-adapting suite of pathogens [28,29], very little is known about the functional differences between AMP alleles in terms of how they interact differentially with both the pathogen and the host [30]. AMPs can be processed into different mature peptide forms that can each have specificity against distinct pathogens, such as the Drosocin peptide in *Drosophila* [31]. Further, previous work with *Drosophila* has indicated that 11 AMPs exhibit trans-species polymorphisms, an indication that alleles may be maintained potentially due to phenotypic differences [30,32,95]. Indeed, functional work on the Imd-regulated Diptericin A peptide indicates that one allele exhibits significantly stronger antimicrobial activity against *Providencia rettgeri* bacterial pathogen challenge [30]. This would indicate that some alleles may be associated with higher host fitness in the presence of specific pathogen infections. Further, as a group, AMPs in *Drosophila melanogaster* and *D*. *mauritiana* show evidence of balancing selection based on calculations of Tajima’s D, π, and Watterson’s θ [33]. These three population genetic measures collectively indicate higher observed levels of AMP nucleotide diversity and intermediate frequency alleles than expected in several populations. Observed nucleotide diversity in AMPs is also higher than that observed across all other immune genes as a group, and across the genome as a whole [33]. These results are consistent with maintenance of AMP variation through balancing selection [33]. Due to the direct interactions of AMPs with pathogens, there is an anticipated evolutionary arms race between AMPs and microbes and an expectation of rapid evolution. In contrast with this expectation, we see maintenance of some alleles rather than rapid changes [34–38]. Indeed, there is growing evidence that AMPs may be maintained via balancing selection in many species, including waterfowl [39], the great tit [40], frogs [41], Persian cats [42], and humans [43]. The trend of balancing selection in AMPs is therefore robustly supported in diverse taxa, and introduces the question of how and why some AMP alleles are balanced in populations of animals such as *Drosophila*.

One intriguing *Drosophila* peptide is Metchnikowin (Mtk), a 26-residue AMP induced by both the Toll and Imd pathways [44,45]. It exhibits activity against a variety of fungi, bacteria, and potentially eukaryotic parasites [14,45–47]. Mtk is expressed in various tissues in response to infection, including the fat body (comparable to human liver), surface epithelia, and the gut [48,49]. It is a proline-rich AMP, others of which are known to inhibit pathogen ribosomes [50]. Functional research in a heterologous yeast system indicates it may interact with pathogen succinate-coenzyme Q reductase or possibly critical fungal cell wall components [51,52]. Mtk may also play a role in the fly nervous system [53,54]. Flies lacking the gene exhibit improved outcomes after traumatic brain injury, indicating that Mtk may have additional functions in the host beyond pathogen defense [53]. These may include roles in sleep and memory functions [55], as well as post-mating responses in females [56]. Importantly, previous work identified two alleles of *Mtk* where the mature peptide has either a proline (*Mtk*^*P*^) or an arginine (*Mtk*^*R*^) in the third to last amino acid position that persist in various populations of *D*. *melanogaster* (80% *Mtk*^*P*^, n = 20), *D*. *simulans* (52% *Mtk*^*P*^, n = 21), *D*. *mauritiana* (20% *Mtk*^*P*^, n = 10), and *D*. *yakuba* (90% *Mtk*^*P*^, n = 20) (data previously reported in Unckless and Lazzaro 2016 [32]). Further data from the PopFly database indicates that populations of *D*. *melanogaster* across the globe have either of the two alleles at varying frequencies (S1 Table). This is despite the fact that species divergence time ranges from an estimated 3–12 million years ago [57,58]. The probability of these same two alleles being found in all four species by neutral processes is therefore exceedingly low. A more plausible explanation is that both alleles play functionally important roles in these populations. Two main hypotheses could account for this repeated functional balance between the two alleles [33]. The *autoimmune hypothesis* posits that one of the alleles has more potent antimicrobial activity, but carries a negative fitness effect in the host in the absence of infection. The expectation with this hypothesis is that flies with this allele would have higher survival with infection, but reduced relative fitness in the absence of infection. Notably, we acknowledge that any negative fitness impact in the absence of infection need not strictly be detrimental effects on the host immune cells, but could be by another mechanism such as detrimental effects on the normal flora of the host. Indeed, flies lacking AMPs or lysozymes exhibit altered microbiomes, indicating their importance in regulating the host microbiota [59]. Throughout, we will broadly define the “autoimmune hypothesis” to encompass negative fitness impacts on either host or microbiota. The *specificity hypothesis* posits that each allele is more potent against a different suite of pathogens. The expectation with this hypothesis is that survival after infection would be determined by the interaction between the allele and specific pathogen, but neither allele would necessarily have a higher fitness cost in the absence of infection. Other hypotheses are also possible, but these two hypotheses are likely and provide a helpful framework for experimental approaches. One long-term outcome of a balanced polymorphism is tandem gene duplication to “fix” both alleles in different paralogs [60,61]. Interestingly, *Mtk* appears to be single copy across much of the *Drosophila* genus although there is an annotated *Mtk-like* gene in *D*. *melanogaster* that is 800kb upstream [62]. The site of the proline/arginine polymorphism in *Mtk* does not have a homologous region in *Mtkl* [63].

Here, we use CRISPR-edited *D*. *melanogaster* strains with either *Mtk*^*P*^, *Mtk*^*R*^, or Mtk* (null) alleles in various life history and infection assays to assess these hypotheses and expectations. We find that in males, the evidence broadly supports the autoimmune hypothesis, where flies with the *Mtk*^*R*^ allele have lower fitness without infection, but generally survive just as well or better than the other alleles when infected. In contrast, the benefit of specific *Mtk* alleles for surviving infection in females is pathogen-dependent. When considering sex by genotype interactions, the evidence therefore also supports the specificity hypothesis, suggesting that both hypotheses may contribute to maintenance of *Mtk* alleles in fly populations.

## Results

### Generation of fly lines with CRISPR-edited Metchnikowin alleles

To assess our two hypotheses, we generated CRISPR-edited *D*. *melanogaster* by injecting Cas9 protein, guide RNA, and a single stranded donor with the desired edit into a *w*^*1118*^ line. This resulted in three replicate lines of *Mtk*^*P*^, *Mtk*^*R*^, and Mtk* alleles (S1 Fig). The three *Mtk*^*P24*^ (henceforth, *Mtk*^*P*^) alleles were derived from siblings of the founders of edited lines, ensuring that they were exposed to the same injection history as the edited lines. This allowed experimentation on three mostly independent *Mtk*^*P*^ strains, three independent *Mtk*^*P24R*^ (henceforth, *Mtk*^*R*^) strains, and three independent Mtk* strains. Notably, the Mtk* strains each have unique deletions in *Mtk*. One has a single base pair (bp) deletion, another a 6 bp deletion, and a third has a 17 bp deletion. Two of these (1bp and 17bp) result in frameshifts, and the other removes two amino acids. Experiments and analyses were performed using the three independent strains of each allele, all experiments were performed with all three strains of all three alleles concurrently (nine total strains side-by-side), and any potential differences among the three strains of an allele type were statistically assessed by including the effect of isoline in statistical models (S2 Table). The isolines grouped together by allele type, both phenotypically and statistically, in the infection experiments. This approach, along with sequencing confirming no off-target effects of CRISPR (S3 Table), ensured results were robustly supported and reduced the likelihood of any results being impacted by other genetic differences. We also performed whole genome resequencing of each line using Illumina short-read sequencing to a depth ranging from 4X to 12X among the lines. Our sequencing approach (S1 Fig and S3 Table) found one missense SNP in *Fen1* (Flap endonuclease 1) that was the reference in all edited lines, but the alternative allele in null and unedited lines. So, while we cannot rule out that off-target effects of CRISPR/Cas9 editing influenced our phenotype, it is likely that the effects observed were due to mutations at *Metchnikowin* and not off-targets.

### *Mtk*^*R*^ allele flies exhibit lower egg hatch rates and die earlier as adults

**Fig 1:**
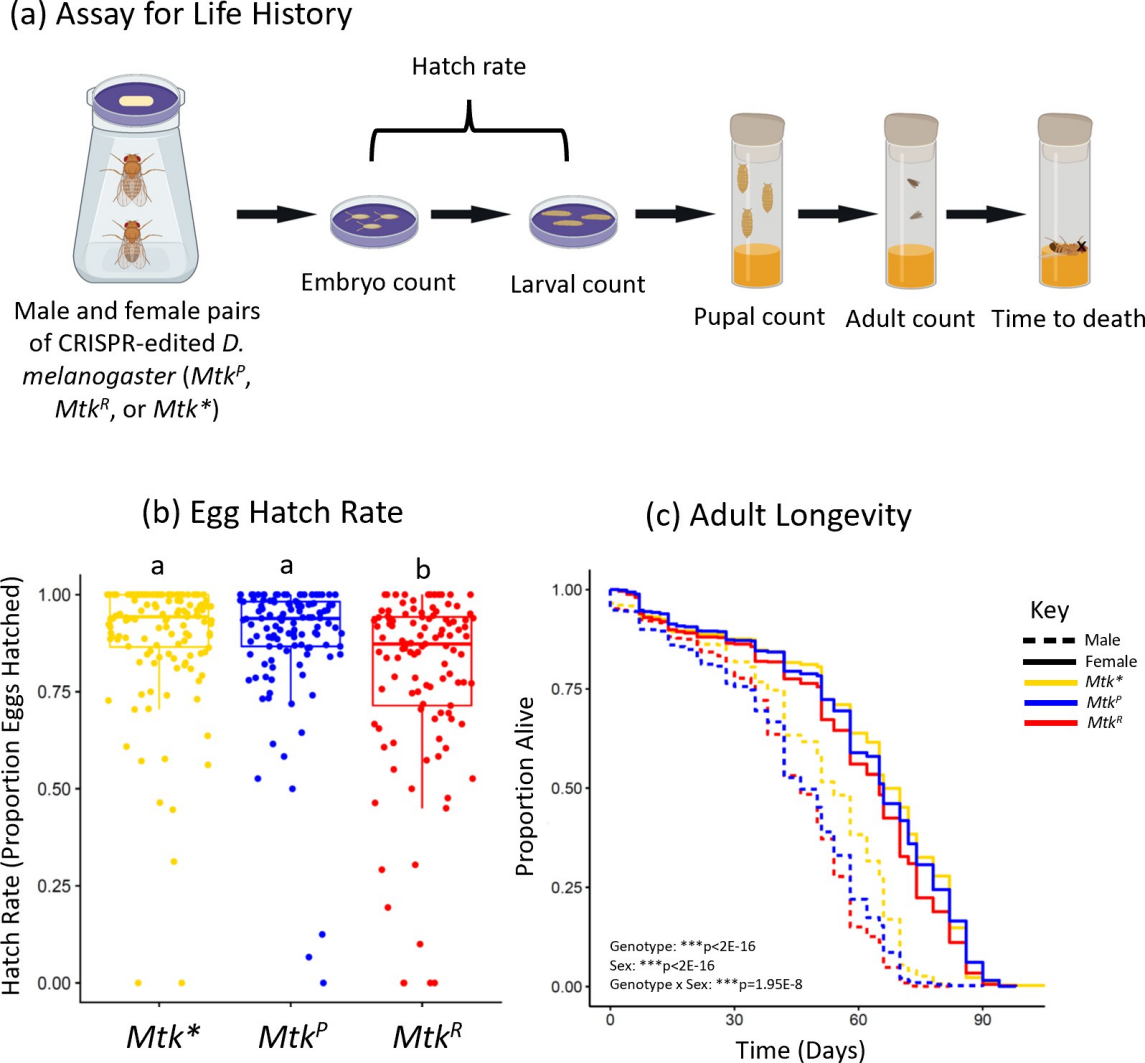
*Mtk*^*R*^ allele flies have lower egg hatch rates and adult longevity times. (a) Overview of the experimental design, where individual pairs of males and females of a given genotype were crossed. They were allowed to lay eggs for 24 hours and those embryos were counted. They were then monitored for hatching rates and the larvae were moved into fly food vials. The pupae were counted each day, along with adult male and female emergence, and those adults were tracked over time until death. Figure was created with BioRender.com. (b) The egg-to-larvae hatch rate was counted for each family. Each dot represents the offspring of a single male and female of the indicated genotype. Each dot represents the proportion of all eggs that hatched from one dish (mean eggs per family was 41). The boxes indicate the interquartile range. Outer edges of the box indicate 25^th^ (lower) and 75^th^ (upper) percentiles and the middle line indicates 50^th^ percentile (median). Whiskers represent maximum and minimum ranges of data within 1.5 times the interquartile range of the box. Letters indicate statistical significance groups, based on a logistic regression and Tukey post hoc test. (c) Lines represent adult longevity beginning at emergence from pupae. Each genotype contains an average of 3879 total flies (males plus females). Statistics based on an ANOVA with Tukey post-hoc test (S2 Table). The entire experiment was performed twice, and the graph represents a combination of data from both experiments.

We performed a life history assay with the *Mtk* strains to assess any fitness differences as anticipated by the autoimmune hypothesis. This assay involved tracking the offspring of 32 individual matings of each genotype from egg to death (Fig 1A). This allowed quantification of offspring survival between life history stages for many families. While egg numbers, survival rates between developmental stages, and the proportion of adult female offspring were similar or exhibited small differences between alleles (S2 Fig and S2 Table), there were a few notable life history differences. The first was the egg-to-larval hatch rate, where approximately 5% fewer *Mtk*^*R*^ eggs hatched compared to *Mtk*^*P*^ or Mtk* (Fig 1B, logistic regression & Tukey contrasts *Mtk*^*R*^ vs *Mtk*^*P*^ or Mtk*, ***p<1x10^-5^). The larval-pupal survival rate was also slightly lower for *Mtk*^*R*^ vs *Mtk*^*P*^ (S2B Fig, logistic regression & Tukey *Mtk*^*R*^ vs *Mtk*^*P*^, *p = 0.04). The second was adult longevity, where *Mtk*^*R*^ flies died slightly but significantly earlier than their counterparts (Fig 1C, ANOVA, ***p_Genotype_<2x10^-16^). *Mtk*^*R*^ males survived 0.44 days less on average than *Mtk*^*P*^ males and 6.04 days less than Mtk* males, while *Mtk*^*R*^ females survived 2.8 days less on average than *Mtk*^*P*^ females and 3.43 days less than Mtk* females. The difference in longevity was thus larger in females between *Mtk*^*R*^ and *Mtk*^*P*^ alleles. In addition, male longevity with *Mtk*^*R*^ varied from experiment to experiment, but earlier female death remained consistent (S3 Fig, ANOVA, Block A ***p_GenotypexSex_ = 4.25x10^-7^, Block B ***p_GenotypexSex_ = 1.28x10^-6^). Third, Mtk* exhibited improved longevity over both alleles, particularly in males (refer to differences in days alive above, Fig 1C, ANOVA and Tukey post hoc test, ***p_R-Null_ = p<2x10^-16^, ***p_P-Null_ = p<2x10^-16^). Overall, these results suggest that *Mtk*, and in particular *Mtk*^*R*^, carries a measurable but small fitness cost in the absence of infection, as expected under the autoimmune hypothesis.

### Male *Mtk*^*R*^ allele flies survive systemic fungal infection as well or better than *Mtk*^*P*^ allele or Mtk* flies

**Fig 2:**
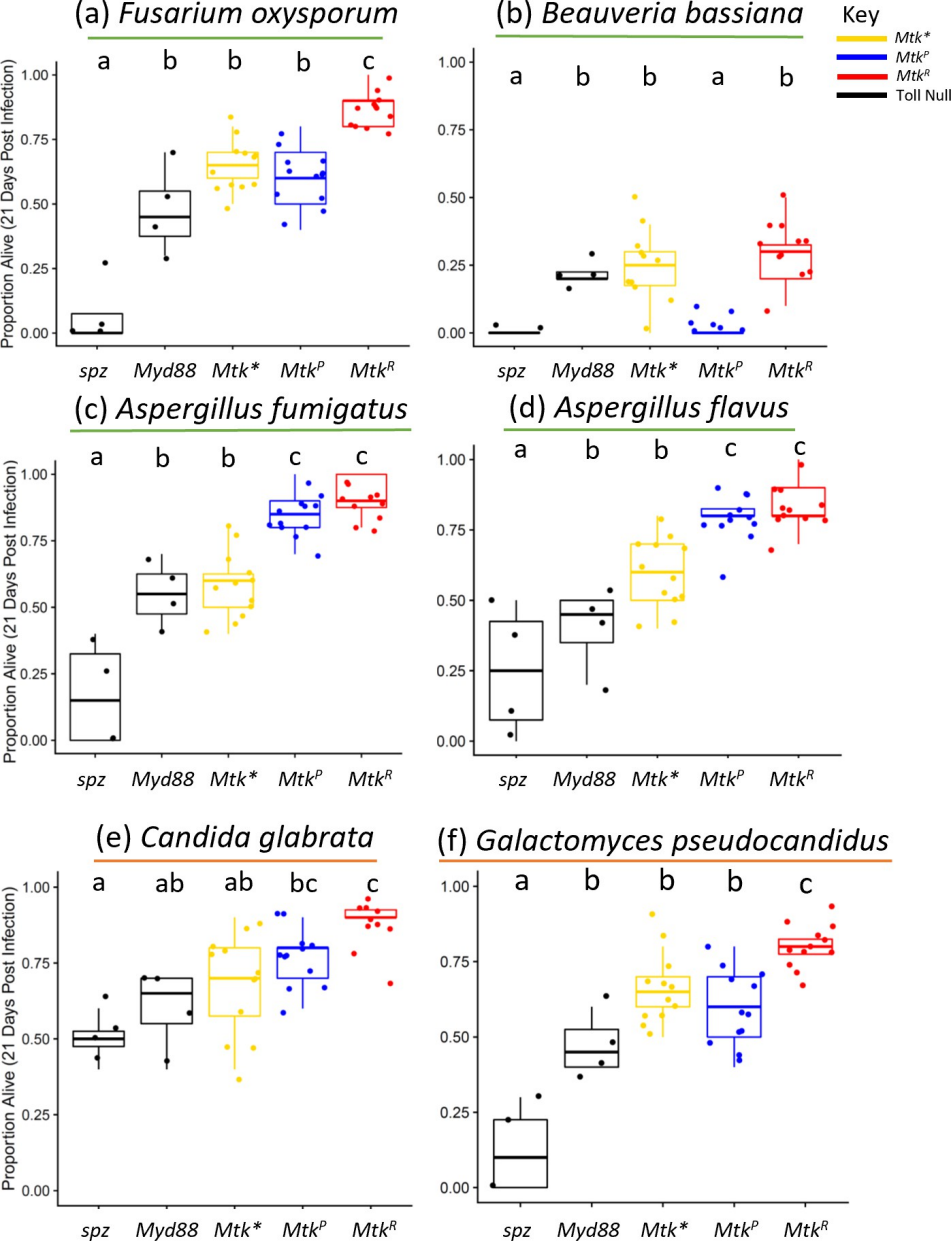
*Mtk*^*R*^ male flies survive as well or better than other genotypes against a variety of filamentous fungal and yeast infections. Infections were performed with the indicated microbes, using either fungal spores (green underline) or yeast cultures (orange underline). (a) *Fusarium oxysporum*, (b) *Beauveria bassiana*, (c) *Aspergillus fumigatus*, (d) *Aspergillus flavus*, (e) *Candida glabrata*, (f) *Galactomyces psuedocandidus*. Each dot represents the survival 21 days after infection for a vial starting with 10 males. Each set of data represents two independent experiments combined. Corresponding survival curves and controls for this experiment are shown in S4 Fig. The boxes indicate the interquartile range. Outer edges of the box indicate 25^th^ (lower) and 75^th^ (upper) percentiles and the middle line indicates 50^th^ percentile (median). Whiskers represent maximum and minimum ranges of data within 1.5 times the interquartile range of the box. Letters indicate statistical significance groups, based on a logistic regression and Tukey post hoc test (S2 Table).

We performed a series of systemic infections in flies by piercing a fungal pathogen-dipped needle into the thorax and measuring survival each day over time to determine whether the different *Mtk* alleles provided different levels of protection against pathogens. We also included 2 additional fly lines that are deficient for either the *spz* or *Myd88* genes to compare loss of *Mtk* to loss of the entire Toll pathway and ensure that infections were resulting in expected levels of death for Toll-deficient lines. We first performed fungal infections in males from lines carrying the different alleles and measured survival over 21 days (Figs 2 and S4). Results revealed that *Mtk* alleles play important roles in the survival outcomes of various infections in males. First, *Mtk* is important for survival after infection with a variety of filamentous fungi and yeast including *Fusarium oxysporum*, *Aspergillus fumigatus*, *Aspergillus flavus*, *Beauveria bassiana*, *Candida glabrata*, and *Galactomyces pseudocandidus*. This result is based on improved survival of flies with either one or both *Mtk* alleles over Mtk* flies. Second, for many pathogens tested, individuals with the *Mtk*^*R*^ allele survived as well or better than those with the *Mtk*^*P*^ allele (Fig 2). Notably, survival of individuals homozygous for *Mtk*^*P*^ was significantly lower than those with the *Mtk*^*R*^ allele after infection with *F*. *oxysporum* (odds ratio (OR): 0.28), *B*. *bassiana* (OR: 0.537), and *G*. *pseudocandidus* (OR: 0.44) (Fig 2 panels a,b,f, logistic regression & Tukey correction *Mtk*^*P*^ vs *Mtk*^*R*^, *F*. *oxysporum* ***p<0.001, *B*. *bassiana* ***p<0.001, *G*. *pseudocandidus* **p = 4.56x10^-3^), suggesting that *Mtk*^*R*^ is more potent at fighting certain fungal infections in *vivo*. Notably in the case of *B*. *bassiana* infection (Fig 2B), the *Mtk*^*P*^ allele was associated with even lower survival than the Mtk* (OR: 0.58), suggesting an unexpected negative interaction between this allele and this pathogen (logistic regression & Tukey correction *Mtk*^*P*^ vs Mtk*, ***p<0.001). In addition, *Mtk*^*P*^ survival was similar to *spz*, and Mtk* survival was similar to *Myd88*, indicating that *Mtk* may have a primary role in immune response to *B*. *bassiana* in *D*. *melanogaster*. These results, in addition to previous results showing a fitness cost with no infection (Fig 1), continue to support the expectations of the autoimmune hypothesis. Another infection, *Candida albicans*, revealed a small but significant difference between alleles with Mtk* flies surviving at the lowest rates (OR: 1.56 vs *Mtk*^*P*^) (S5A Fig).

### *Mtk* allelic variation is associated with differential survival in males after infection with some bacterial pathogens

**Fig 3:**
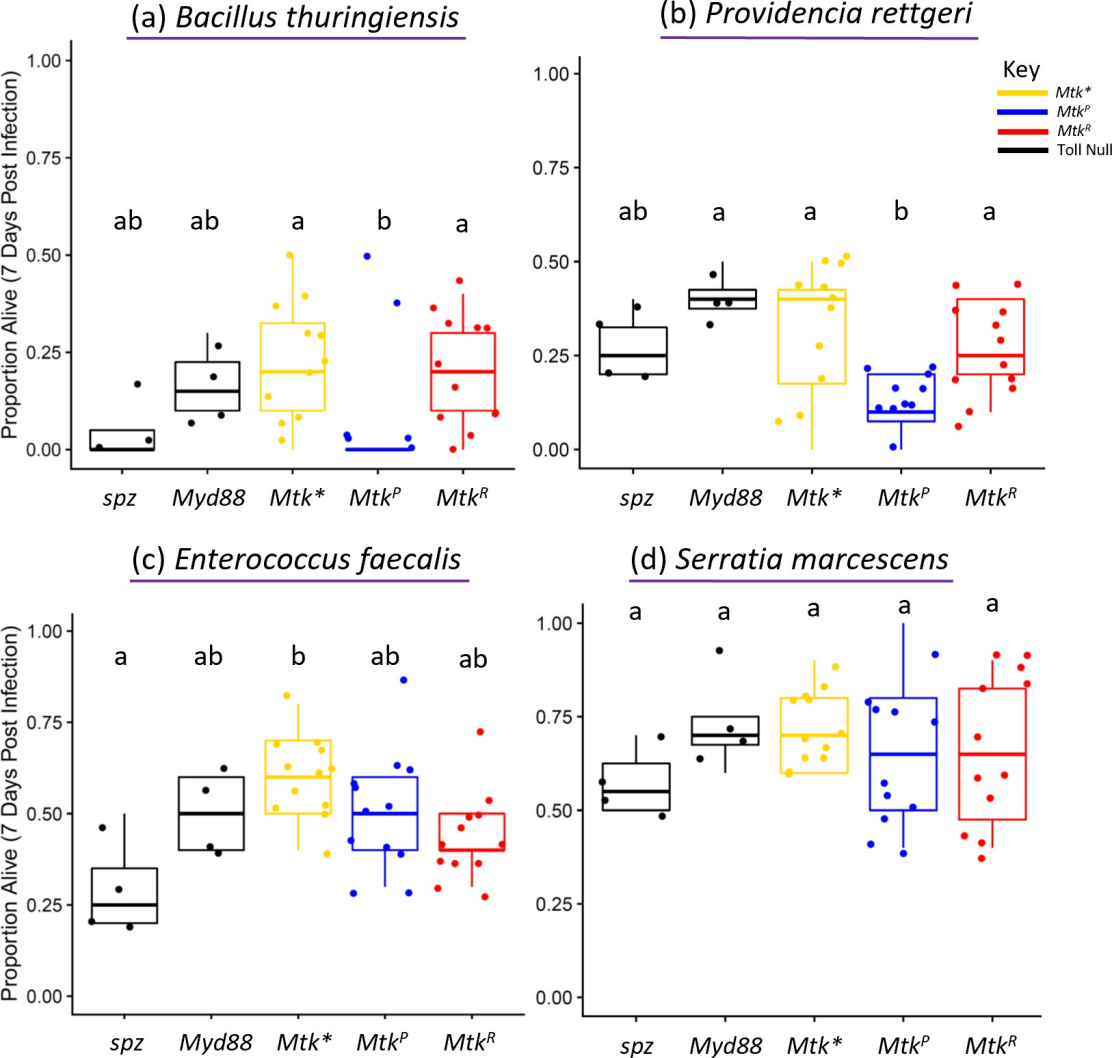
*Mtk* alleles are associated with differential survival after infection with some bacterial infections in males. Infections were performed with the indicated microbes, using bacteria (purple underline). (a) *Bacillus thuringiensis*, and (c) *Enterococcus faecalis*, are Gram-positive. (b) *Providencia rettgeri*, and (d) *Serratia marcescens*, are Gram-negative. Each dot represents the survival 7 days after infection for a vial starting with 10 males. Each set of data represents two independent experiments combined. Corresponding survival curves and controls for this experiment are shown in S6 Fig. The boxes indicate the interquartile range. Outer edges of the box indicate 25^th^ (lower) and 75^th^ (upper) percentiles and the middle line indicates 50^th^ percentile (median). Whiskers represent maximum and minimum ranges of data within 1.5 times the interquartile range of the box. Letters indicate statistical significance groups, based on a logistic regression and Tukey post hoc test (S2 Table).

Metchnikowin is canonically expressed in response to both fungal and bacterial pathogens, and was initially characterized with potent *in vitro* activity against both *Micrococcus luteus* and *Neurospora crassa* [45]. To determine whether *Mtk* genotype is associated with differential ability to survive bacterial infection, we performed additional systemic infections in males using a variety of Gram-positive bacteria (*Bacillus thuringiensis*, *Enterococcus faecalis*, *Lysinibacillus fusiformis*, *Staphylococcus succinus*, *Staphylococcus sciuri*, *Lactococcus brevis*, and *Lactococcus plantarum*) (Figs 3A, 3C, S5B–S5F, S6A and S6C), since the Toll pathway partially controls *Mtk* expression and canonically responds to Gram-positive bacteria and fungi. We also tested two Gram-negative bacteria (*Providencia rettgeri* and *Serratia marcescens*) for comparison (Figs 3B, 3D, S6B and S6D). We found that while many bacterial infections result in no differences in survival across *Mtk* alleles (Figs 3C and 3D and S6), both *B*. *thuringiensis* and *P*. *rettgeri* infections did (Fig 3A and 3B). *Mtk*^*P*^ flies survived at lower rates than *Mtk*^*R*^ flies (logistic regression & Tukey correction *Mtk*^*R*^ vs *Mtk*^*P*^, *B*. *thuringiensis* *p = 4.45x10^-2^ & OR: 0.73, *P*. *rettgeri* *p = 3.11x10^-2^ & OR: 0.72). Notably and similar to the results with *B*. *bassiana* infection, both of these bacterial infections resulted in reduced survival of the *Mtk*^*P*^ allele compared to Mtk* (OR: 0.62 *B*. *thuringiensis*, OR: 0.62 *P*. *rettgeri*). This result again suggests there is a negative interaction between the *Mtk*^*P*^ allele and certain pathogen infections in male flies.

### There is an interaction between sex and *Mtk* allele for survival after infection

**Fig 4:**
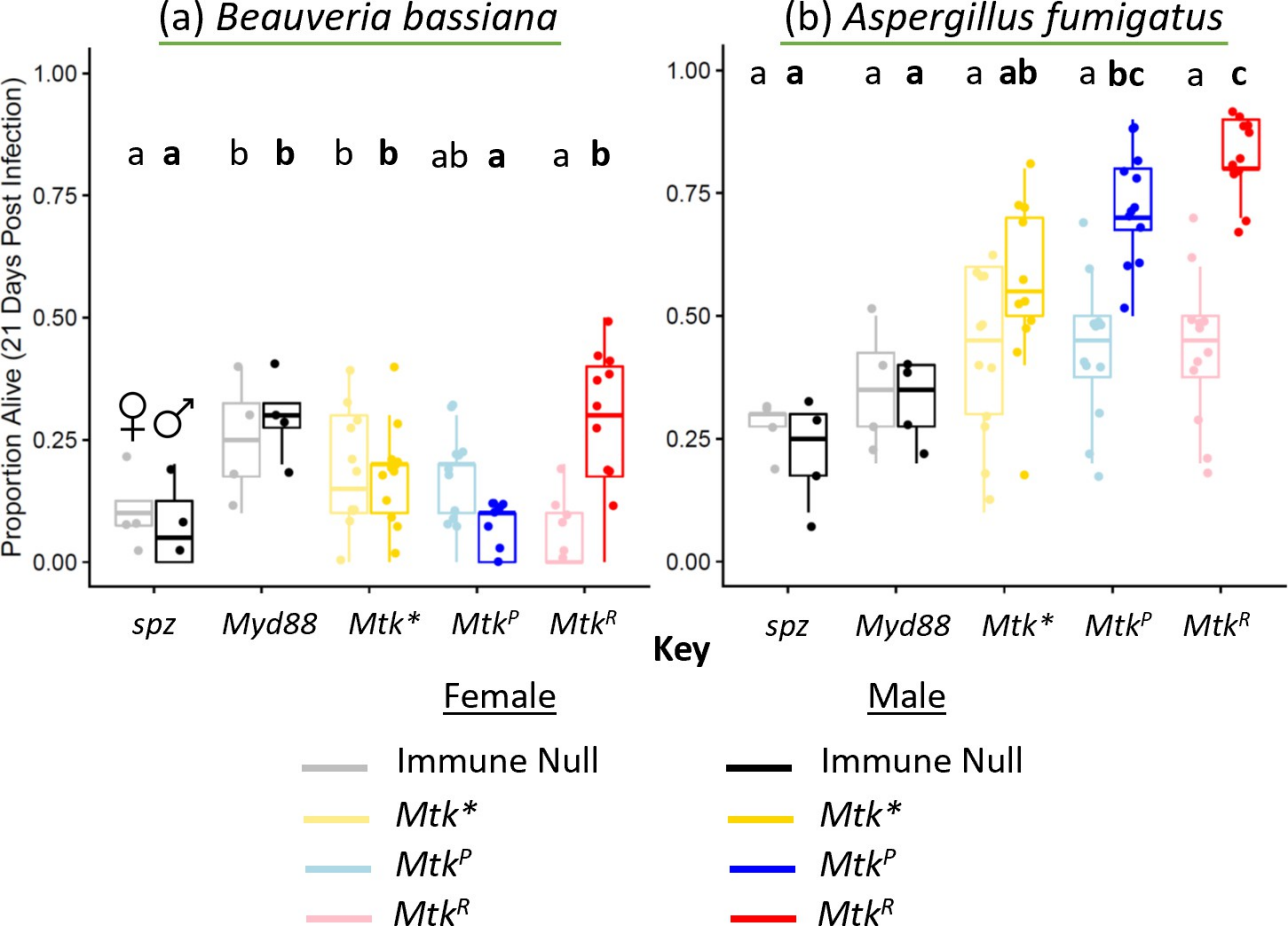
Side-by-side male and female fungal spore infections demonstrate key differences in phenotypes. Infections were performed in females and males with the indicated microbes, using two representative sporulating fungal species (green underline). (a) *Beauveria bassiana*, (b) *Aspergillus fumigatus*. Each dot represents survival 21 days after infection for a vial starting with 10 flies. Each set of data represents two independent experiments combined. Females are shown in the lighter colors on the left of each genotype pair, and males are shown in the darker colors on the right of each genotype pair. Corresponding survival curves and controls for this experiment are shown in S9 Fig. The boxes indicate the interquartile range. Outer edges of the box indicate 25^th^ (lower) and 75^th^ (upper) percentiles and the middle line indicates 50^th^ percentile (median). Whiskers represent maximum and minimum ranges of data within 1.5 times the interquartile range of the box. Letters indicate statistical significance groups, based on logistic regression and Tukey post-hoc test (S2 Table). Non-bolded letters indicate females, and bolded letters indicate males.

Differences between male and female innate immune functions are common [64–68]. As such, we also performed systemic infections with females and a subset of representative bacterial and fungal pathogens to get a preliminary idea of any potential sex by genotype interactions (S7 and S8 Figs). Based on results of systemic infection experiments in males, we chose three pathogens that produced significant differences in survival between *Mtk* alleles (*P*. *rettgeri*, Gram-negative bacteria; *B*. *bassiana*, sporulating fungus; and *C*. *glabrata*, yeast) and two pathogens that showed no difference between *Mtk*^*P*^ and *Mtk*^*R*^ alleles (*E*. *faecalis*, Gram-positive bacteria; *A*. *fumigatus*, sporulating fungus). Only a subset of pathogens was tested in females since only a subset is needed to answer the question of whether there are differences in survival between the sexes with respect to *Mtk*. Compared to male infections, several key differences were found. First, *Mtk* alleles were not associated with survival after infection with *P*. *rettgeri* or *A*. *fumigatus* in females, which was not true for males. Second, females infected with *B*. *bassiana* had higher survival rates with the *Mtk*^*P*^ allele than the *Mtk*^*R*^ allele (OR: 1.4, logistic regression & Tukey correction *Mtk*^*P*^ vs *Mtk*^*R*^, **p = 2.33x10-3). This is in direct contrast to the result for males, where the opposite was true. A more direct side-by-side comparison of male and female survival differences was then performed using infections with *A*. *fumigatus* (*Mtk* allele important in males but not females) and *B*. *bassiana* (opposite survival between the sexes). This confirmed the differing phenotypes (Figs 4 and S9), where specific *Mtk* allele was an important determinant of survival in male *A*. *fumigatus* infection but not in females (ANOVA *Mtk*^*P*^ & *Mtk*^*R*^ only, ***p_Sex_ = 8.31x10^-15^), and where the *Mtk*^*R*^ allele was less beneficial than *Mtk*^*P*^ for females (OR: 0.65) and the *Mtk*^*P*^ allele is less beneficial for males than *Mtk*^*R*^ (OR: 0.5) in *B*. *bassiana* infection (ANOVA *Mtk*^*P*^ & *Mtk*^*R*^ only, ***p_GenotypexSex_ = 5.2x10^-8^). These results suggest a more nuanced version of the pathogen specificity hypothesis where sex and pathogen may interact to determine infection outcomes for different AMPs.

### Flies carrying the *Mtk*^*R*^ allele are more resistant to infection than *Mtk*^*P*^

**Fig 5:**
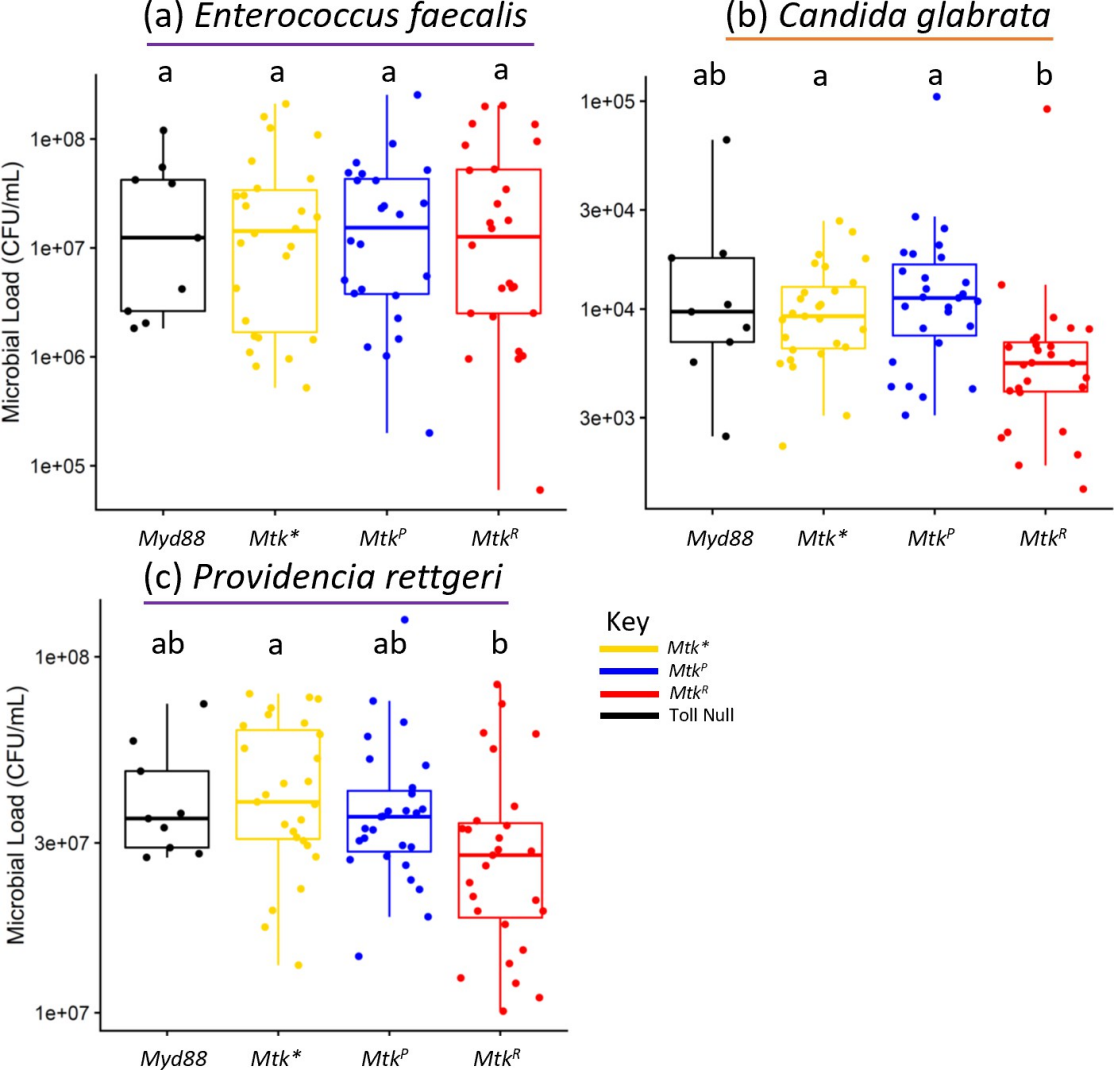
Pathogen load differences suggest that greater pathogen resistance may underlie higher survival of *Mtk*^*R*^ males with certain infections. Infections were performed in males with the indicated microbes, using bacteria (purple underline) or yeast (orange underline). (a) *Enterococcus faecalis*, (b) *Candida glabrata*, (c) *Providencia rettgeri*. Each dot represents pathogen load 24 hours after infection for 3 pooled flies. Each set of data represents three independent experiments combined. The boxes indicate the interquartile range. Outer edges of the box indicate 25^th^ (lower) and 75^th^ (upper) percentiles and the middle line indicates 50^th^ percentile (median). Whiskers represent maximum and minimum ranges of data within 1.5 times the interquartile range of the box. Letters indicate statistical significance groups, based on a logistic regression and Tukey post hoc test (S2 Table).

To assess whether allelic differences in survival are based on pathogen tolerance (host is infected but less symptomatic) or resistance (host reduces pathogen burden) [69], we measured microbial load in males infected with a subset of bacteria and yeast that exhibited no allelic effects (*E*. *faecalis*), higher *Mtk*^*R*^ survival (*C*. *glabrata*), or *Mtk*^*P*^ survival below that of Mtk* (*P*. *rettgeri*) (Fig 5). In general, results were consistent with *Mtk* alleles playing a role in resistance to certain infections. *Mtk*^*R*^ flies had a significantly lower pathogen loads after *C*. *glabrata* infection compared to both *Mtk*^*P*^ (15% fewer colony forming units) and Mtk* flies (40% fewer colony forming units) (logistic regression & Tukey correction, Mtk* & *Mtk*^*R*^ *p = 3.79x10^-2^, *Mtk*^*P*^ & *Mtk*^*R*^ **p = 3.02x10^-3^), and significantly lower pathogen loads than Mtk* flies after *P*. *rettgeri* infection (30% fewer colony forming units, logistic regression & Tukey correction, **p = 5.7x10^-3^). However, we also found that pathogen loads after *P*. *rettgeri* infection were not significantly different between *Mtk*^*P*^ and Mtk* flies, despite *Mtk*^*P*^ files exhibiting lower survival (Fig 3B). No pathogen load differences were observed for flies of different *Mtk* genotypes infected with *E*. *faecalis*, as expected given survival after infection with this pathogen was not influenced by *Mtk* genotype (Fig 3C). These results suggest that *Mtk*^*R*^ may increase pathogen resistance and therefore survival in males, but that pathogen load does not underlie lower survival with *Mtk*^*P*^ vs Mtk* alleles after *P*. *rettgeri* infection.

### *dnaK* is unlikely to play a role in allelic differences in survival

As *dnaK* has been previously implicated as a potential microbial target for another proline-rich AMP [70], we infected our CRISPR-edited *Mtk* lines with a *dnaK* deletion strain of *E*. *faecalis* to assess if loss of this putative target would result in differential survival among flies of different *Mtk* alleles. However, whether infected with the deletion strain or the parent strain (intact *dnaK*), survival was no different across alleles (S10 Fig).

### *Mtk* is expressed in wild-type flies without infection and CRISPR fly strains express all alleles at similar RNA transcript levels

The public database FlySexsick-seq is a transcriptomic resource to compare sexually dimorphic gene expression in Canton S wild-type flies 8 hours post-infection with *P*. *rettgeri* or with no microbial challenge [71]. The results show that *Mtk* baseline expression in males is present and is about 4.5% of the expression level with infection, while female baseline expression is about 5.5% of the expression level with infection (S11A Fig). Thus, though there is strong induction after infection, there is also a measurable expression level without any infection. Further, we performed qPCR to measure *Mtk* gene expression levels in our CRISPR lines with or without *B*. *bassiana* infection (S11B Fig). We find similar results, in that non-infected flies have a low baseline level of expression without infection. Male baseline expression ranges from 0.3–5.0% of the expression level with infection (depending on genotype), and female baseline expression ranges from 0.3–15% of the expression level with infection. The differences are largely based on wide variance in expression levels with infection, where female *Mtk*^*P*^ and *Mtk*^*R*^ flies have the highest *Mtk* expression with infection, with males at 9.35% and 28.4% of female expression levels, respectively.

## Discussion

Animals must maintain a delicate balance with their immune systems to maximize survival during infection while reducing any off-target damage to themselves. AMPs are a group of innate immune peptides that play a crucial role in host immune responses to infection in a wide variety of species. Prior work in *Drosophila* has indicated that AMPs might be under balancing selection, which has been postulated to mediate this balance between host fitness and antimicrobial activity [33]. Indeed, several specific AMPs have been identified where multiple alleles are maintained in fly populations across the globe despite expectations of rapid evolution [30,32]. However, there have been few investigations either empirically validating allelic functional differences or identifying mechanisms underlying cases of likely balancing selection [30,33]. The two main hypotheses for AMP allelic diversity maintenance are the autoimmune hypothesis (one allele is costly without infection, but beneficial with infection) and the specificity hypothesis (each allele is more effective against a different suite of pathogens). There are other hypotheses as well, which are not yet ruled out, but these two are likely and provide a helpful framework to guide initial experiments. Here, with CRISPR/Cas9-edited fly strains, we show strong *in vivo* evidence of functional differences between two AMPs that differ by a single amino acid. We find results consistent with the autoimmune hypothesis, with the *Mtk*^*R*^ allele exhibiting greater antimicrobial activity than *Mtk*^*P*^ in many contexts while simultaneously carrying a host fitness cost. However, when incorporating host sex, a more complex picture arose, where sex interacted with host genotype in predicting survival after infection with some pathogens. Broadly, the results the results support a pleiotropic model where host genotype, sex, pathogen species, and infection status collectively and differentially impact host survival outcomes, rather than proving one hypothesis over another. We term this the ‘context-dependent’ hypothesis. Future work will be required to more fully assess each individual hypothesis and to rule out other hypotheses, but the results so far are consistent with the autoimmune and specificity hypotheses to different degrees.

The life history assay revealed several points of interest regarding the relative roles of *Mtk* alleles in flies (Fig 1). First, egg hatch rate was lower in flies with the *Mtk*^*R*^ allele. This fitness cost would support the autoimmune hypothesis. Since lower egg hatch occurred in the absence of infection and during the embryonic development stage, this may indicate a role of *Mtk* outside of pathogen response in a process that is important in early development. Alternatively, it could indicate baseline expression that has off-target effects in the host. Second, there were differences in longevity among genotypes and between the sexes. For example, the *Mtk*^*R*^ allele had a higher fitness cost for both sexes, but more so in females relative to *Mtk*^*P*^. Also, Mtk* flies of both sexes lived longest in both sexes, but the difference was greater in males. These results may shed light on *Mtk* function and fitness costs. The fact that the Mtk* allele flies of both sexes have the highest survival demonstrates that the *Mtk* gene carries a fitness cost in the absence of infection, regardless of sex. Mtk* allele males had a larger increase in longevity relative to the other alleles, and that the difference is higher than that in females, indicating a higher cost of *Mtk* in males. Reasons for this are not yet known, but may indicate that *Mtk* alleles have sex-biased functions or expression, or an off-target effect that is particularly harmful to males. Notably, there is some baseline transcriptional expression of *Mtk* without infection, with results from the FlySexsick-seq database indicating that male *Mtk* expression is about 4.5% of the expression level with *P*. *rettgeri* infection, while female baseline expression is about 5.5% compared to expression levels with infection (S11A Fig). Similar results were found in qPCR data from *B*. *bassiana* infection in the lines tested in this study, where baseline expression was low but present (S11B Fig). This baseline level of expression suggests *Mtk* could have a function beyond response to infection and that lower fitness without infection could be due to negative effects of baseline *Mtk* expression on either the host or normal flora. Additionally, the lower egg hatch results indicate a potential role of *Mtk* in processes outside of pathogen responses that may be important in both embryos and adults. Or, baseline expression may be harmful at both stages. Finally, the fact that *Mtk*^*R*^ displayed the most significantly reduced longevity in the absence of infection, along with lower egg hatch rates, supports the autoimmune hypothesis. The reasons for these sex differences are unclear and will need further study. For example, they could be due to differences in food consumption, where females might eat more due to their high reproductive output and incidentally encounter more pathogens and require *Mtk* more often. Alternatively, sex differences could be due to a role of *Mtk* in immune responses to mating or reproduction [55], or other functions in the host unelated to pathogen responses that may differ between sexes such as microbiome regulation.

Infections in males also revealed some interesting patterns. Notably, *Mtk*^*R*^ flies outlive *Mtk*^*P*^ flies with some infections (Figs 2 and 3). This might be due to stronger activity against certain pathogens. In other cases, both alleles resulted in similarly higher survival compared to Mtk* flies, indicating that while *Mtk* expression is important in surviving these infections, the R/P polymorphism does not play a role in infection outcome. In still other cases, both the *Mtk*^*R*^ and Mtk* flies unexpectedly had higher survival than *Mtk*^*P*^ flies. In this last case, we postulate that Mtk might interact with the pathogen in some complex way, such that the P form of the allele enhances mortality. This may be via pleiotropic functions of Mtk in the brain [53,55,56], or through negative regulatory or direct interactions with secreted fungal molecules, which can occur with Mtk and other AMPs [72,73]. This result suggests that host outcomes depend on multiple factors including pathogen identity and host genotype, and that the exact mechanism of functional differences may not simply be specific alleles being more potent against specific microbes. We term this complex interplay between host genotype, host phenotype and pathogen the ‘context-dependent hypothesis.’

Male infection experiments indicate that a wide variety of fungi and bacteria are inhibited by *Mtk* (Figs 2 and 3). Expanding on prior results, we show that *Mtk* expression is important in defense against various filamentous fungal, yeast, and bacterial infections. Notably, others have shown that microbes including *Micrococcus luteus* [45], *Neurospora crassa* [45], and *Fusarium graminaerum* [52], are inhibited by *Mtk* in vitro or in heterologous systems. In addition, several studies show that *Mtk* expression is induced upon infection with microbes including *Beauveria bassiana* [14,74,75], while others do not identify *Mtk* upregulation with infection [76]. Our results support the role of *Mtk* in *Beauveria bassiana* infection in vivo and expand on previous findings by demonstrating allelic differences in survival. Indeed, we found that homozygosity for the *Mtk*^*P*^ allele was worse than not expressing *Mtk* at all, being as detrimental to survival after *B*. *bassiana* infection as knocking down expression of the entire Toll pathway (Fig 2B). Additionally, we find evidence that other microbes are inhibited by this AMP: filamentous fungi *Aspergillus fumigatus*, *Aspergillus flavus*, and *Fusarium oxysporum*, yeasts *Candida glabrata* and *Galactomyces pseudocandidus*, and bacteria *Bacillus thuringiensis* and *Providencia rettgeri*. We found a small but significant role of *Mtk* in *Candida albicans* infection (S5A Fig) as previously reported [46]. Thus, there is agreement with prior work on the role of *Mtk* in fighting a broad range of microbial infections in several genera. We also found no evidence of a role of *Mtk* in response to infections with *Enterococcus faecalis*, *Serratia marcescens*, *Lysinibacillus fusiformis*, *Staphylococcus succinus*, *Staphylococcus sciuri*, *Lactococcus brevis*, and *Lactococcus plantarum* infections. Thus, we find a few patterns. One is that *Mtk* exhibits activity against most filamentous fungi tested, several yeasts, and only a small subset of mostly Gram-positive bacteria. Another is that while Mtk is active against Gram-positive bacteria, as previous studies have indicated [45], there may be some activity against Gram-negative bacteria as well, and a role of *Mtk* in fighting Gram-negative bacteria is a novel discovery. A final point is that many of the microbes that are not inhibited by *Mtk* include members of the host microbiome (in particular, *Lactococcus* species that were isolated from flies). There are many possible reasons for this, including that these strains are simply not as pathogenic as the others and did not elicit strong enough immune responses, when used in our systemic infection studies, to reveal differences between alleles. Alternatively, Mtk may not impact the normal flora negatively as it does with pathogens for unknown reasons. Given that Mtk is expressed in the trachea, surface epithelia, and gut in addition to the fat body [48,49], it is possible that normal flora are not impacted by Mtk, or are differentially impacted. Importantly, *Drosophila* AMPs including Mtk are collectively important for regulating the host microbiome [59].

Female infection experiments are also revealing (Figs 4 and S7). In some cases, results are similar between the sexes, such as with *Candida glabrata* (where both alleles help fight infection) and *E*. *faecalis* (no role of *Mtk*). In other cases, specific *Mtk* allele matters in males (*P*. *rettgeri* and *A*. *fumigatus*), but not females. Finally, for the most lethal pathogen used in this study, *B*. *bassiana*, the *Mtk*^*P*^ is beneficial and *Mtk*^*R*^ detrimental in females, while the opposite is true in males. Though this result was replicated across experiments for both sexes (Figs 2 and 5 and S7), the effect size is somewhat small and future work to assess this conclusion using independent methods will be important, such as overexpressing the alleles in each sex to determine if that rescues the survival differences. This will be important to validate the subtle sex by genotype interaction reported in these fly lines. However, and interestingly, the qPCR expression results (S11B Fig) demonstrated some differences in *Mtk* transcription between the sexes upon infection with *B*. *bassiana* that may partially underlie the differences in infection outcomes between the sexes (Fig 4A). Indeed, sex is a significant variable in determining *Mtk* expression level (ANOVA, * p_sex_ = 0.014). Although there are similarly low baseline expression levels between males and females of all genotypes in the control treatments, the expression of *Mtk* in females infected with *B*. *bassiana* is significantly higher than males. Thus, transcriptional-level differences in early infection may be an important contributing factor to the differences in infection outcomes between the sexes. A prior study indicated that Toll pathway signaling underlies some sexual dimorphism in infection outcomes, with relative differences between the sexes in infection outcomes changing in Toll mutants through a mechanism that is not yet fully understood^71^. However, few samples were tested thus far for *Mtk* expression (S11B Fig), so further testing and analysis will still be necessary to confirm these differences and investigate the underlying mechanism. While insignificant in females, a similar pattern of allelic differences in survival was found for males with *P*. *rettgeri*, another very pathogenic infection in this host. This could suggest that there are sex-specific benefits to each *Mtk* allele that are only manifested in severe infections, which likely elicit the strongest immune responses. It is still unclear why these differences may exist, although it does suggest that *Mtk* expression overall, and by allele, can have remarkably different functions between the sexes that significantly affect survival and fitness. This result could explain why *Mtk* is maintained as a balanced polymorphism in *D*. *melanogaster*. Future work will be necessary to determine the mechanism behind sex-based differences. For example, differential regulation of alleles may occur due to reproductive-immune tradeoffs, or Mtk may regulate the microbiome in functionally important and sex-dependent ways. Future work will also be required to determine why, in some cases, one allele not only performs worse than the other, but also worse even than Mtk*. Notably, previous work has demonstrated a sexual dimorphism in *D*. *melanogaster* survival with *B*. *bassiana* infection, with females being more susceptible due to differences in immune pathway signaling [77] and due to factors including mating status and diet [78]. This difference based on mating status might be an important factor to test in the future with the lines in this study, as sex differences may be driven in part by female mating status. Loss-of-function mutations in Toll pathway or *relish* (an *Imd* gene) removes the dimorphism [77]. Likewise, we found no differences between the sexes in survival in *Toll*-deficient and Mtk* flies (Figs 4 and S9), and a sexual dimorphism in flies expressing *Mtk*, mediated by allelic identity with this infection. However, it is still unclear why either allele can be actively detrimental in certain conditions, and future work will be needed to assess this further.

We also took initial steps to assess the mechanism of the generally more potent *Mtk*^*R*^ activity and found that microbial suppression likely underlies it (Fig 5 and S2 Table). There was lower microbial load in *Mtk*^*R*^ flies infected with *C*. *glabrata*, and a similar (insignificant) trend in *P*. *rettgeri*. In contrast, *E*. *faecalis*, which was not impacted by *Mtk* allele, showed no difference in pathogen load. These results mostly parallel the infection survival assays and indicate that *Mtk*^*R*^ is either actively killing or inhibiting growth of pathogens as a part of its mechanism. Future experiments will be needed to assess whether these pathogen load differences are due to expression differences, different binding capacities, or other differences relating to the microbial target. Finally, we found no evidence for a role of *dnak* being an important microbial target, as with or without the gene, *E*. *faecalis* infection resulted in similar survival rates for hosts with different *Mtk* alleles (S10 Fig). Currently, the microbial molecular target of Mtk is unclear. Previous studies that attempted to identify a pathogen target were based on experiments in a heterologous yeast system with filamentous fungal targets and results have not been confirmed in a natural system or through other methods [51,52]. It is possible that Mtk may have different mechanisms of antimicrobial activity in fungi vs bacteria.

Here we present evidence of several new aspects of Mtk biology: 1) *Mtk* alleles are functionally distinct. 2) Differential fitness in the context of host *Mtk* allele, host sex, infection status, and pathogen identity is likely to underlie *Mtk* allele maintenance in wild fly populations. 3) Differences in host survival can be mediated by even a single amino acid polymorphism in a single AMP. 4) A wide range of pathogen species, including novel species shown in this study, are impacted by *Mtk* expression. 5) Host sex plays a critical role in the outcome of infection, and this interacts with *Mtk* allele. 6) The autoimmune hypothesis was broadly supported: *Mtk*^*R*^ appears to be more potent in many infectious contexts with a fitness cost in the absence of infection. However, some contexts of varying host sex and pathogen identity provide some support to the specificity hypothesis. Thus, we suggest a new ‘context-dependent’ hypothesis that incorporates both prior hypotheses in different contexts. 7) Finally, further work to elucidate the sex-based differences in *Mtk* allele outcomes along with other aspects of *Mtk* biology, such as its microbial target and putative non-pathogen response host functions, would be valuable.

## Materials and methods

### Identification of allele frequencies in *D*. *melanogaster* populations

The PopFly database [79] was used to retrieve 20 bases around the P/R SNP for 761 inbred lines from 30 different *D*. *melanogaster* populations around the globe. For each population, the number of G bases (R allele), C bases (P allele), and N bases (no base called), were counted and the total proportion of R alleles was then calculated (S1 Table). Notably, R allele frequencies are highest in Africa, absent in many populations except North America, and in Africa are higher at higher elevations and latitudes.

### Generation and validation of CRISPR fly strains

An inbred *w*^*1118*^ stock was used as the wildtype line for CRISPR/Cas9 genome editing. Cas9 protein, guide RNA (gRNA), and single-stranded donor DNA containing the appropriate edits (S1 Fig) was injected. Lines were created with the intended edit (a single nucleotide mutation resulting in a change from proline to arginine in the 24^th^ residue of the mature Mtk peptide as well as linked silent mutations to mutate the PAM sites) via homology directed repair, null alleles resulting from nonhomologous end joining, and unedited *Mtk* alleles carried by flies that had been subject to the same injections as the edits and the nulls (S1 Fig). Two gRNAs (Mtk_gRNA_target6 and Mtk_gRNA_target14) were designed with PAM sequences just upstream of the desired edit site and synthesized using the New England Biolabs EnGen sgRNA Synthesis Kit, *S*. *pyogenes* Protocol (New England Biolabs, Ipswich, MA, USA). A 120 bp single stranded donor oligo (Mtk_ssDNA1) was used with the desired edit (changing the proline codon–CCA to an arginine codon–CGA, as well as editing the PAM site to protect against further edits) (Eurofins, Fisher Scientific LLC, Chicago, IL, USA). The gRNAs, single stranded donor oligo, and Cas9 (PNA Biosciences Cas9 Protein with NLS, NC1279639, Fisher Scientific) were injected into 240 embryos per gRNA by GenetiVision Corporation (Houston, TX, USA).

Several of the injected embryos did not survive to adulthood, but those that did were mated to the parent *w*^*1118*^ line and their offspring were Sanger sequenced using primers Mtk_F1 and Mtk_R1 (S4 Table, includes all primers for all experiments including CRISPR and qPCR [80], described below) as a brute force approach to finding edits. Any F1 individuals with evidence of edits (double peaks around the edit site) were inbred to isolate and create homozygous mutants. Siblings of the putative edited individuals were kept as unedited controls. Through several generations of crosses, homozygous lines (confirmed by Sanger sequencing, S1 Fig) were created with the edit (3 lines that are likely 3 independent edits since they were derived from different G0 embryos) or deletions (1bp, 6bp or 17bp), as well as 3 unedited control lines (S1 Fig). Note that the first number (152 in Edit_Arginine_152.5.6.3.1) refers to the initial line derived from a single initial embryo injection so most lines within an allele were derived from different embryos (though two of the edits were derived from the same embryo).

To ensure that these lines didn’t significantly differ in sequence other than the focal edits in *Mtk*, we performed light whole genome sequencing on each of the nine lines derived from *w*^*1118*^. Briefly, we extracted DNA from each line using the Qiagen Gentra Puregene Tissue Kit (158066, Qiagen, Germantown, MD, USA), then prepared library using the Illumina Nextera DNA Library Preparation Kit (Illumina, Inc., San Diego, CA, USA). Samples were sequenced on a fraction of a HiSeq2000 lane to an average depth ranging from 4 to 12. As expected, the number of pairwise differences between any of our CRISPR/Cas9 genome edited lines was low (less than 200 putative differences per chromosome).

Even though the total number of pairwise differences between lines was low, this does not rule out off-target effects that preferentially effect one genotype class (null vs. unedited vs. edited). We therefore annotated all 158 SNPs that were fixed between genotype class (S6 Table). Of those, we chose 14 to investigate further because they were either in genes that had a known role in the immune system, or were likely functional changes (missense, UTR, etc.). S6 Table shows that of the 14 SNPs examined in detail, only one actually appears to be different between genotype classes. That SNP is a missense mutation in *Fen1* that is the reference “T” in edited lines and the alternative “A” in null and unedited lines. *Fen1* encodes Flap endonuclease 1, which is involved in DNA replication and repair [63]. Although *Fen1* has no annotated immune function, we cannot rule out that it plays some role in the phenotypic differences observed. All other SNPs that we investigated were clearly cases of ambiguous mapping with individuals called as “ref” or “alt” clearly carrying both “ref” and “alt” alleles. This contrasts with the SNPs and deletions in *Mtk* that were unambiguously different between strains.

Finally, although the *Mtk* guide RNAs were selected because they were predicted to have no off-target effects, we interrogated for off-targets by blasting the two guide RNAs against the *D*. *melanogaster* genome using blastn-short. This yielded one perfect match for each guide RNA (the intended targets) and several other matches that matched at best 15 bases (we cut used a length cutoff of at least 14 bases of match). Of the 11 matches we found, only one had any SNPs in the vicinity of the target and that one was an intronic SNP in *CG32264* (involved in actin binding) which was the “alt” allele in all lines except one of the unedited lines.

We therefore, interrogated for off target of our genome editing in two ways: first by searching for evidence of SNPs and other mutations in the sequenced lines, and second by looking at putative off-target sites. In neither case do we find compelling evidence for off target effects that are likely to influence immune defense or other traits. Instead, most of the segregating sites we examined carefully appear to be errors in sequencing, mapping or variant calling that are inevitable in this size of a data set. However, given the nature of the problem, we cannot rule out the possibility that off-target effects influence the phenotypes observed. It is also important to acknowledge that our approach cannot rule out that the silent changes caused by mutated the PAM site might influence our phenotypes, but given that these are silent and we see limited differences in expression between alleles, this seems unlikely.

### Fly strains and husbandry

Additional fly strains included *spz* (*spz/rm7*, loss of function point mutation [81], gift from B. Lazzaro), and *Myd88* (BDSC 14091, transposable element insertion). Flies were reared on CMY media: 64.3 g/L cornmeal (Flystuff Genesee Scientific, San Diego CA), 79.7 mL/L molasses (Flystuff Genesee Scientific), 35.9 g/L yeast (Genesee Scientific inactive dry yeast nutritional flakes), 8 g/L agar (Flystuff Genesee Scientific *Drosophila* type II agar), 15.4 mL of antimicrobial mixture [50 mL phosphoric acid (Thermo Fisher, Waltham MA), 418 mL propionic acid (Thermo Fisher), 532 mL deionized water], and 1g/L tegosept (Genesee Scientific). Flies were reared, mated, and housed after infection, at room temperature (25°C) and were kept on a 12 h light/dark light cycle.

### Microbial strains and growth conditions for fly infections

The microorganisms used in this study are summarized in S5 Table. To grow cultures for fly infections, bacteria and yeast isolates were grown for 16 h from a single colony in 2 mL media with shaking at 225 rpm. Most bacteria were grown at 37°C in Luria broth (LB) (Teknova, Hollister CA) from single colonies on Luria agar (LA) (molecular biology grade agar, Teknova) and yeast were grown at 30°C in potato dextrose broth (PDB, BD, Sparks MA) from colonies on potato dextrose agar (PDA). *Lactococcus* species were grown in MRS broth (BD) from single colonies on MRS agar plates at 30°C. Fungal spores were prepared by purifying spores grown at 30°C on PDA for 1–2 weeks. Autoclaved DI water was poured over each plate and the spores were suspended in the liquid. This was then poured over a filter (Millipore Sigma, Burlington MA, Miracloth 22–25 μm pore size) and the filtrate was placed into a 50 mL falcon tube. This was then centrifuged at 1000 rpm for 5 min and the supernatant was discarded. The spores were then resuspended in sterile 20% glycerol and were counted using a hemocytometer and stored at 4°C until use. Fungal concentrations were as follows (conidia/mL): *Aspergillus fumigatus* (1.75x10^9^), *Aspergillus flavus* (1.18x10^8^), *Fusarium oxysporum* (9.65x10^7^), and *Beauveria bassiana* (4.38x10^8^).

### Fly infections

Bacteria and yeast were grown overnight in the conditions described above. Yeasts *C*. *glabrata*, *C*. *auris*, *C*. *albicans*, and *G*. *pseudocandidus* were diluted in PDB to an optical density (OD) value of A_600_ = 35 +/- 1 for *Candida glabrata* and *Candida auris*, an OD value of A_600_ = 250 +/- 5 for *Candida albicans*, and an OD value of A_600_ = 120+/- 1 for *Galactomyces*. Bacteria were diluted in LB to an OD value of A_600_ = 4 +/- 0.02 for *B*. *thuringiensis*, *E*. *faecalis*, *L*. *brevis*, *L*. *plantarum*, and *S*. *marcescens*, an OD value of A_600_ = 1.5 +/- 0.02 for *E*. *faecalis* K-12 and Δ*dnaK*, and an OD value of A_600_ = 1 +/- 0.02 for *P*. *rettgeri*. Sporulating fungi were prepared as described above. Males or females 4–6 days old of a given genotype were pierced in the thorax just beneath the wing using a 0.15 mm dissecting pin (No. 15 Minuten pins, 12 mm long, 0.15 mm diameter, Entosphinx, Czech Republic) dipped into the diluted culture or control. Controls were the growth broth for yeasts and bacteria (PDB or LB, see above) or sterile 20% glycerol for the fungal spores. Flies were then placed in groups of 10 per food vial. 20 individuals of each treatment x sex x isoline group were infected in each block (3 isolines per genotype), and at least two blocks of infections were performed on separate days for every experiment. Flies infected with fungi were counted daily for survival for 21 days and those infected with bacteria were counted for 7 days, as differences in survival across treatment groups were observed within these time periods. Infection data for survival over time was analyzed by a COX proportional hazard model with proportion dead as the dependent variable and *Mtk* allele (R, P, or Null) and infection day as independent variables, with the specific isoline within the *Mtk* allele as a random variable (results of full model available in S2 Table corresponding to the full experimental datasets over time corresponding to S4–S10 Figs). For the graphs depicting only the endpoint survival rates, we used a simpler ANOVA model with proportion alive as the dependent variable, and infection day and allele as independent variables.

### Life history assay

Virgin females of each isoline of the *Mtk* CRISPR flies were collected and aged 2–4 days. They were then crossed to males of the same isoline, also 2–4 days old. This was done by placing single male-female pairs of each isoline into a 6 oz. square bottom *Drosophila* bottle (Fisher Scientific, Hampton NH) covered with a grape juice agar plate [100% concord grape juice (Welch’s, MA), tegosept (Genesee Scientific), absolute ethanol (Decon Laboratories Inc, PA), agar (Teknova, Hollister CA), DI water] with yeast paste (Fleischmann’s Active Dry Yeast, Heilsbronn Germany). There were 32 crosses per isoline for the first block and 16 crosses per isoline for the second. These bottles were placed in a 25°C incubator overnight (12-hour dark/light cycle). Grape plates were swapped the next morning (16 hr later) with fresh plates and yeast. The bottles were placed back in the incubator and flies were allowed to lay eggs for 24 h. Then, plates were removed and adults discarded. The number of eggs was immediately counted and the plates were placed back into the incubator. The number of hatched embryos was counted the next day and larvae were moved into vials with CMY *Drosophila* media and the vials were kept in the incubator. Both pupae and emerged adult offspring were counted. The adult offspring were separated by sex into groups of 20 and placed in fresh CMY food vials and kept in the incubator. Flies were given fresh food vials every 4–7 days. Surviving fly numbers were recorded daily until fly death. Longevity data was analyzed by ANOVA and Tukey post-hoc test with lifespan (days alive) as the dependent variable and *Mtk* allele (R, P, or null) and experiment block as independent variables and specific isoline within *Mtk* allele as a random variable. Note that this is a departure from infection survival analysis where we used Cox Proportional Hazard models. The ANOVA approach was used here because all individuals died making the censoring functions of the Cox Proportional Hazard approach unnecessary. We favor the simpler statistical model when possible. Other life history plots (dot plots for egg lay, egg hatch, larval-pupal survival, and pupal-adult survival) were analyzed using an ANOVA and Tukey post-hoc comparison with those factors as dependent variables and *Mtk* allele, isoline/strain, and experimental block as independent variables. The adult sex ratio was analyzed the same way, with sex as an additional interacting factor.

### Microbial load assay

Microbes were grown overnight in 2 mL culture vials with shaking at 225 rpm. Bacteria *E*. *faecalis* and *P*. *rettgeri* were grown in LB at 37°C. Yeast *C*. *glabrata* was grown in PDB at 30°C. After 16 h growth, the cultures were diluted to the desired OD values: A_600_ = 4 +/- 0.02 for *E*. *faecalis*, A_600_ = 1 +/- 0.02 for *P*. *rettgeri*, and A_600_ = 35 +/- 1 for *C*. *glabrata*. Infections were performed as described above, by piercing flies in the thorax with a needle dipped in culture or control. For each isoline x treatment combination, nine 3–4 day old males were infected. The flies were kept at room temperature (25°C) for 24 hr, then divided into 1.7 mL Eppendorf tubes with 3 groups of 3 flies for each isoline x treatment combination. Then, 150 μL sterile PBS (Corning, Corning NY) was added to each tube, along with a single sterile 2 mm glass bead (Merck, Germany), and flies were homogenized in a bead beater (Hard Tissue Homogenizer, VWR, Radnor PA) for 3 minutes at 1600 rpm. The homogenates were then placed on ice and 50 μL was plated on either LBA (bacteria or LB controls) or PDA (yeast or PDB controls) using a Whitley WASP Touch Automated Spiral Plater (Don Whitley Scientific, West Yorkshire, UK). The plates were incubated overnight (37°C for LBA plates, 30°C for PDA plates) and then counted using a colony counter (IUL Flash & Go Automated Colony Counter, Neutec Group Inc., Farmingdale NY). One caveat is that 0 h measurements were not taken to measure load in flies at the beginning of infection, however, this technique does typically give reproducible microbial inoculums to flies [82] and the three replicates of this experiment showed similar trends in microbial loads. Microbial load was analyzed using an ANOVA and Tukey post-hoc test with microbial load (CFU/mL) as the dependent variable, *Mtk* allele and experimental block as independent variable, and isoline as a nested variable within *Mtk* allele.

### qPCR gene expression assay

For each isoline, 3 flies of each sex were infected with *B*. *bassiana* or not infected (controls) as described above. This led to 36 total samples (9 isolines, 2 treatments, 2 sexes), with 3 flies per sample/replicate. Flies were kept at 25°C for 16 h and frozen at -80°C until processing. RNA was then extracted using the Qiagen Rneasy kit (cat. 74104) according to the manufacturer’s instructions while using the optional Qiagen Rnase-free Dnase kit (cat. 79254) to treat samples with Dnase per the manufacturer instructions. cDNA was then generated using the Bio-Rad iScript cDNA synthesis kit (cat. 170–8891) using the manufacturer protocol. qPCR was run using Bio-Rad Sso-Advanced Universal SYBR Green Supermix (cat. 1725271) using the manufacturer protocol with *rp49* and *Mtk* primers listed in S4 Table. qPCR was performed on a Bio-Rad CFX Connect Real-Time System. Conditions were as follows: 50°C 10 min, 95°C 5 min, 40x (95°C 10 s, 55°C 30 s), 95°C 30 s. Differences in gene expression were measured by calculating 2^-Δct^. The data was analyzed using an ANOVA using 2^-Δct^ as the dependent variable and *Mtk* allele, sex, and treatment as independent variables, and isoline as a nested variable within *Mtk* allele.

### Data and statistical analyses

Details on statistical models are given within their relevant materials and methods sections. Data analysis and figure generation were performed in R [83] v4.2.2, using several packages: ggplot2 [84] (version 3.4.0), cowplot [85] (version 1.1.1), car [86] (version 3.1.1), SurvMiner [87] (version 0.4.9), MultComp [88] (version 1.4.20), ggExtra [89] (version 0.10.0), ggtext (version 0.1.2) [90], emmeans (version 1.8.3) [91], stringr (version 1.5.0) [92], and SurvMisc [93] (version 0.5.6). All data including numerical data underlying graphs, statistical outputs, and code is available through Dryad at https://doi.org/10.5061/dryad.kkwh70sb5 [94].

## Dryad DOI

10.5061/dryad.kkwh70sb5

## Supporting information

S1 Fig Schematics of CRISPR-generated *Mtk* fly lines. (a) The *w*^*1118*^ sequence represents the starting sequence of the background line (P allele). Two guide RNAs (gRNA) were used to generate flies with the R allele (ssDNA1). The bottom 5 lines depict the nucleotide and amino acid sequences for the lines used in this paper. (b) Electropherograms of sequencing from all 9 total isolines used in the study (segment shown contains all SNPs in this gene between lines). All three *Mtk*^*R*^ lines (edit lines) have the same sequences as each other, as do the *Mtk*^*P*^ lines (unedit lines). Each null allele line has its own unique sequence as shown.
(TIF)

S2 Fig Offspring numbers, survival from larvae to adults, and the proportion of F1 females exhibit small to no differences among *Mtk* alleles. (a) The number of offspring was counted for each family. Each dot represents all eggs from one female (overall mean of 37 eggs per sample/dot). (b) The larvae-to-pupae survival rate was counted for each family. Each dot represents all pupae from offspring of a single female, with an average of 29 pupae per sample. (c) The pupae-to-adult survival rate was counted for each family (average of 27 adults per sample). (d) The proportion of adult offspring that were female compared to total adult offspring (average of 27 adults total per sample, with an average of 14 males and 13 females). Outliers are largely due to families with few offspring. Box plots for each genotype are plotted to the right of the scatter plot for comparison among genotypes (no significant differences). For all graphs, each dot represents the offspring of a single male and female of the indicated genotype. The boxes indicate the interquartile range. Outer edges of the box indicate 25^th^ (lower) and 75^th^ (upper) percentiles and the middle line indicates 50^th^ percentile (median). Whiskers represent maximum and minimum ranges of data within 1.5 times the interquartile range of the box. Letters indicate statistical significance groups, based on a logistic regression and Tukey post hoc test (S2 Table). Families that laid no eggs were not included (usually due to death of a fly during the experiment; similar number of families excluded across alleles). The entire experiment was performed twice, and graphs represent a combination of data from both experiments.
(TIF)

S3 Fig The longevity of adult male *Mtk*^*R*^ varies, but *Mtk*^*R*^ females consistently die earlier. (a) The longevity of adult offspring in block A, with an average of 2182 flies per genotype, sexes combined. (b) The longevity of adult offspring in block B, with an average of 1697 flies per genotype, sexes combined. Statistics based on an ANOVA with Tukey post-hoc test (S2 Table). The experiment was performed twice.
(TIF)

S4 Fig Survival curves for males infected with fungi, corresponding to Fig 2. Infections were performed with the indicated microbes, using either spores (green underline) or yeast cultures (orange underline). Each line represents the survival of 120 flies (*Mtk* alleles and controls) or 40 flies (*spz* and *Myd88*) over a 21-day period for the same data graphed in Fig 2. Statistics based on Cox proportional hazard model (S2 Table). The experiment was performed twice, with combined results represented here.
(TIF)

S5 Fig Survival curves for males infected with bacteria or yeast. Infections were performed with the indicated microbes, using bacteria (purple underline) or yeast (orange underline). Each line represents the survival of 120 flies (*Mtk* alleles and controls) or 40 flies (*spz* and *Myd88*) over a 7-day period. Statistics based on Cox proportional hazard model (S2 Table). The experiment was performed twice, with combined results represented here.
(TIF)

S6 Fig Survival curves for males infected with bacteria, corresponding to Fig 3. Infections were performed with the indicated microbes, using bacteria (purple underline). Each line represents the survival of 120 flies (*Mtk* alleles and controls) or 40 flies (*spz* and *Myd88*) over a 7-day period for the same data graphed in Fig 3. Statistics based on Cox proportional hazard model (S2 Table). The experiment was performed twice, with combined results represented here.
(TIF)

S7 Fig Survival against some infections depends on host sex along with *Mtk* allele and pathogen species. Infections were performed in females with the indicated microbes, using bacteria (purple underline), fungal spores (green underline), or yeast (orange underline). Each dot represents survival 21 days after infection for a vial starting with 10 females. Each set of data represents two independent experiments combined. Corresponding survival curves and controls for this experiment are shown in S8 Fig. The boxes indicate the interquartile range. Outer edges of the box indicate 25^th^ (lower) and 75^th^ (upper) percentiles and the middle line indicates 50^th^ percentile (median). Whiskers represent maximum and minimum ranges of data within 1.5 times the interquartile range of the box. Letters indicate statistical significance groups, based on a logistic regression and Tukey post hoc test (S2 Table).
(TIF)

S8 Fig Survival curves for females infected with bacteria, fungal spores, or yeast corresponding to S7 Fig. Infections were performed with the indicated microbes, using bacteria (purple underline), fungal spores (green underline), or yeast (orange underline). Each line represents the survival of 120 flies (*Mtk* alleles and controls) or 40 flies (*spz* and *Myd88*) over a 21-day period. Statistics based on Cox proportional hazard model (S2 Table). The experiment was performed twice, with combined results represented here.
(TIF)

S9 Fig Survival curves for side-by-side male and female fungal spore infections corresponding to Fig 4. Infections were performed with the indicated microbes, using fungal spores (green underline). Each line represents the survival of 120 flies (*Mtk* alleles and controls) or 40 flies (*spz* and *Myd88*) over a 21-day period. Statistics based on Cox proportional hazard model, with false discovery rate corrections for a subset of contrasts (S2 Table). The experiment was performed twice, with combined results represented here.
(TIF)

S10 Fig Males infected with *dnaK*-deficient *E*. *faecalis* (Δ*dnak*) survive at similar rates across alleles. Infections were performed with *E*. *faecalis* (purple underline). Each line represents the survival of 120 flies (*Mtk* alleles and controls) or 40 flies (*spz* and *Myd88*) over a 21-day period. Statistics based on Cox proportional hazard model (S2 Table). The experiment was performed twice, with combined results represented here.
(TIF)

S11 Fig *Mtk* is expressed at low baseline levels without infection and is highly induced with infection. (a) Graph of *Mtk* expression levels (TPM, transcripts per million reads) in male and female Canton S flies either unchallenged with infection (UC) or challenged with *Providencia rettgeri*, 8 hours post-infection. Data from FlySexsick-seq database^71^. (b) qPCR data of *Mtk* gene expression with or without infection across CRISPR lines used in this study. Each data point represents a pool of 3 flies (whole bodies) either infected with *Beauveria bassiana* or not infected (control). qPCR based on levels of *Mtk* gene expression vs host *rp49* housekeeping gene expression. Values denote 2^-(Δct)^. Statistics are based on logistic regression and Tukey post-hoc test (S2 Table).
(TIF)

S1 Table Proportion of R alleles in various *D*. *melanogaster* fly populations on the PopFly Database. (CSV)

S2 Table Complete statistical analysis outputs for all figures. (XLSX)

S3 Table Pairwise SNP differences among whole genomes (2L, 2R, 3L, 3R and X) of CRISPR/Cas9 genome edited lines and three random DGRP lines. (DOCX)

S4 Table Oligos used in CRISPR/Cas9 editing of *Drosophila Mtk* alleles and qPCR. (DOCX)

S5 Table Microorganisms used in this study. (DOCX)

S6 Table Potential off-targets from CRIPSR. "potential_offtargets" tab shows all 158 SNPs that were "fixed" in one genotype compared to the others. Columns are chromosome, position, ref and alternative alleles (based on dm6.54) and the genotype calls for all 9 sequenced lines. The info column is the output from snpEff, the "fixed" column describes which Mtk genotype was different from the others, and the "NearestGenes" column lists the actual gene symbol affected. Rows in yellow were selected for further analysis because they were putatively functional SNPs or in immune-related genes. The next several tabs show the data for individual SNPs. Each tab has a small table with basic SNP annotations and a screen shot from IGV. Most of these show that the alleles are segregating within line even though they were called as ref or alt. The "Mtk SNPs" tab shows the IGV output for the region containing Mtk mutations. The "Predicted_Offtargets" tab shows the regions of the genome that had blast homology to the two gRNAs (at least 14bp match). This tab is standard blastn output except the final column which suggests that very few of these potential off targets had any SNPs.
(XLSX)
