# Supplementary figures and images for: A single amino acid polymorphism in natural Metchnikowin alleles of Drosophila results in systemic immunity and life history tradeoffs
Source: PLoS Genet. 2024 Mar 11;20(3):e1011155. doi: 10.1371/journal.pgen.1011155 (PMC10957085; doi:10.1371/journal.pgen.1011155)

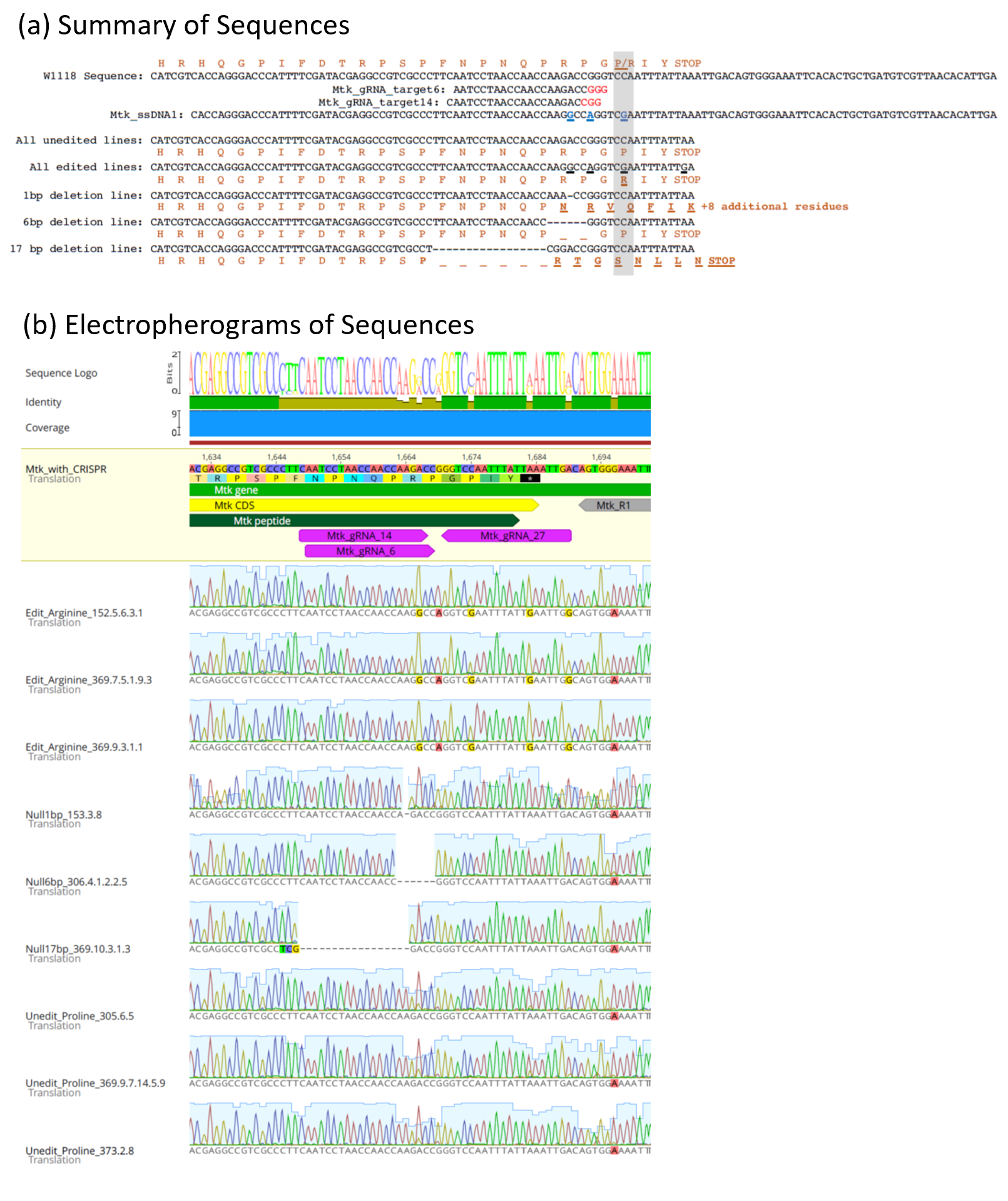

Supplement: S1 Fig — (a) The w1118 sequence represents the starting sequence of the background line (P allele). Two guide RNAs (gRNA) were used to generate flies with the R allele (ssDNA1). The bottom 5 lines depict the nucleotide and amino acid sequences for the lines used in this paper. (b) Electropherograms of sequencing from all 9 total isolines used in the study (segment shown contains all SNPs in this gene between lines). All three MtkR lines (edit lines) have the same sequences as each other, as do the MtkP lines (unedit lines). Each null allele line has its own unique sequence as shown. (TIF) [file pgen.1011155.s001.tif]

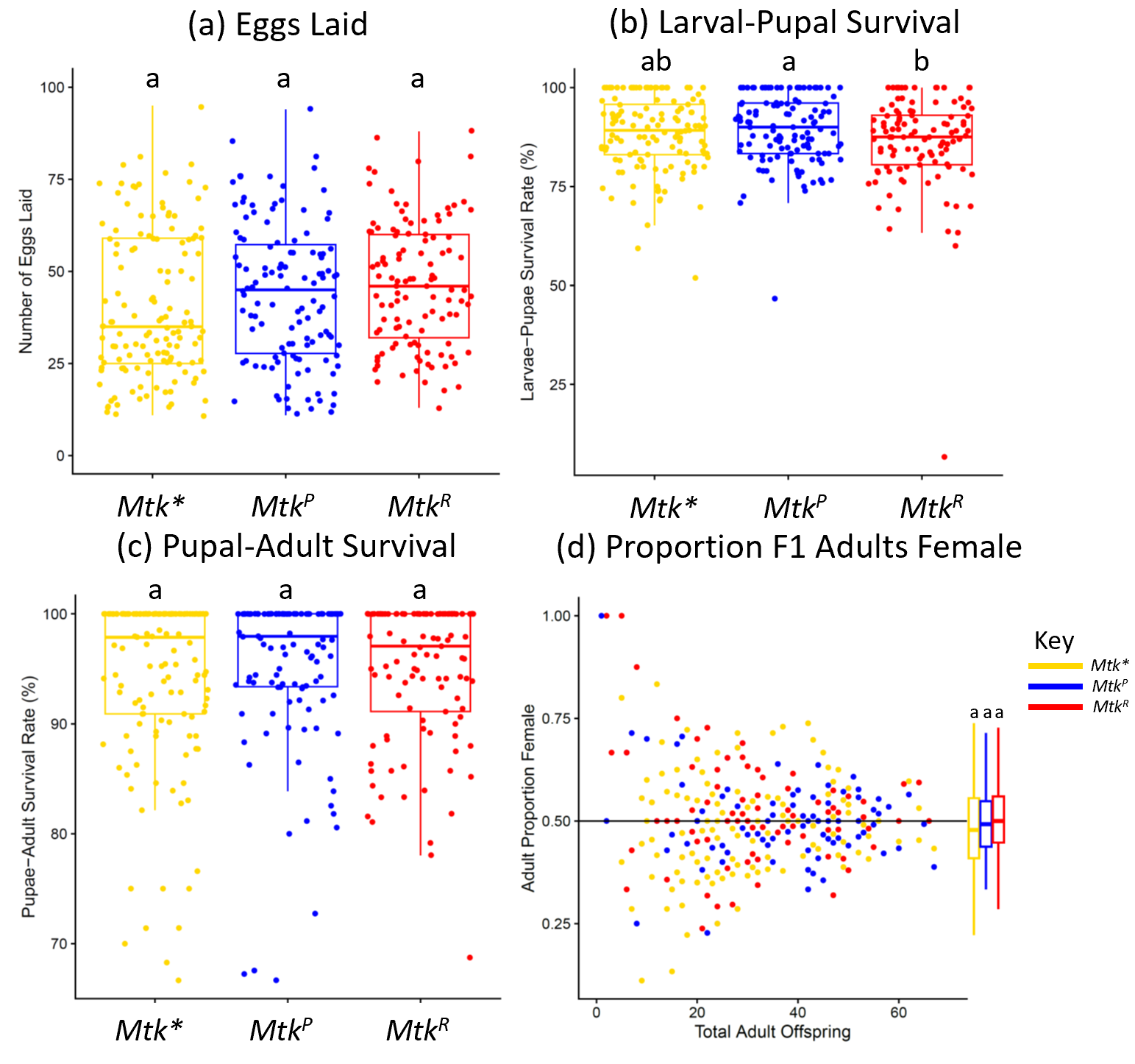

Supplement: S2 Fig — (a) The number of offspring was counted for each family. Each dot represents all eggs from one female (overall mean of 37 eggs per sample/dot). (b) The larvae-to-pupae survival rate was counted for each family. Each dot represents all pupae from offspring of a single female, with an average of 29 pupae per sample. (c) The pupae-to-adult survival rate was counted for each family (average of 27 adults per sample). (d) The proportion of adult offspring that were female compared to total adult offspring (average of 27 adults total per sample, with an average of 14 males and 13 females). Outliers are largely due to families with few offspring. Box plots for each genotype are plotted to the right of the scatter plot for comparison among genotypes (no significant differences). For all graphs, each dot represents the offspring of a single male and female of the indicated genotype. The boxes indicate the interquartile range. Outer edges of the box indicate 25th (lower) and 75th (upper) percentiles and the middle line indicates 50th percentile (median). Whiskers represent maximum and minimum ranges of data within 1.5 times the interquartile range of the box. Letters indicate statistical significance groups, based on a logistic regression and Tukey post hoc test (S2 Table). Families that laid no eggs were not included (usually due to death of a fly during the experiment; similar number of families excluded across alleles). The entire experiment was performed twice, and graphs represent a combination of data from both experiments. (TIF) [file pgen.1011155.s002.tif]

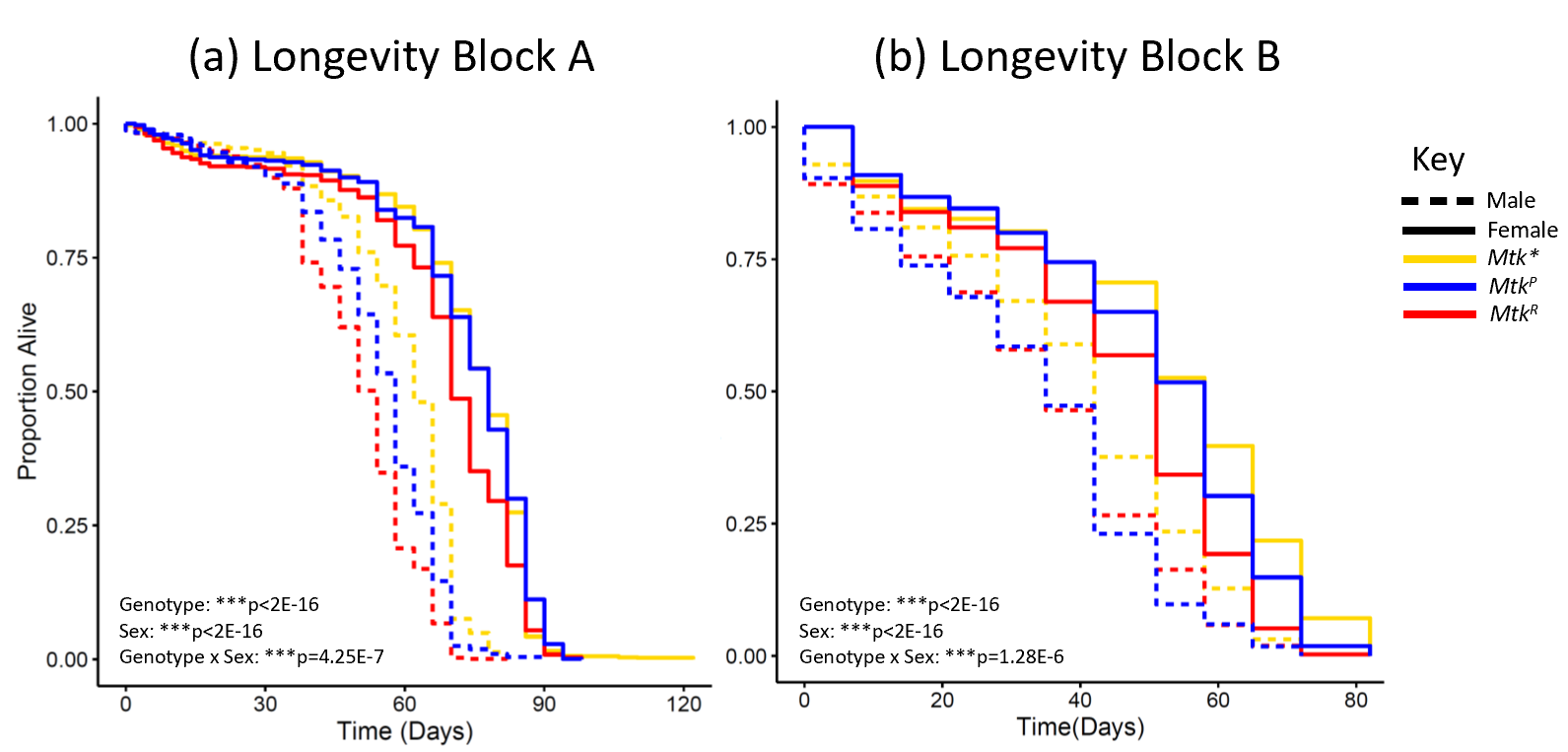

Supplement: S3 Fig — (a) The longevity of adult offspring in block A, with an average of 2182 flies per genotype, sexes combined. (b) The longevity of adult offspring in block B, with an average of 1697 flies per genotype, sexes combined. Statistics based on an ANOVA with Tukey post-hoc test (S2 Table). The experiment was performed twice. (TIF) [file pgen.1011155.s003.tif]

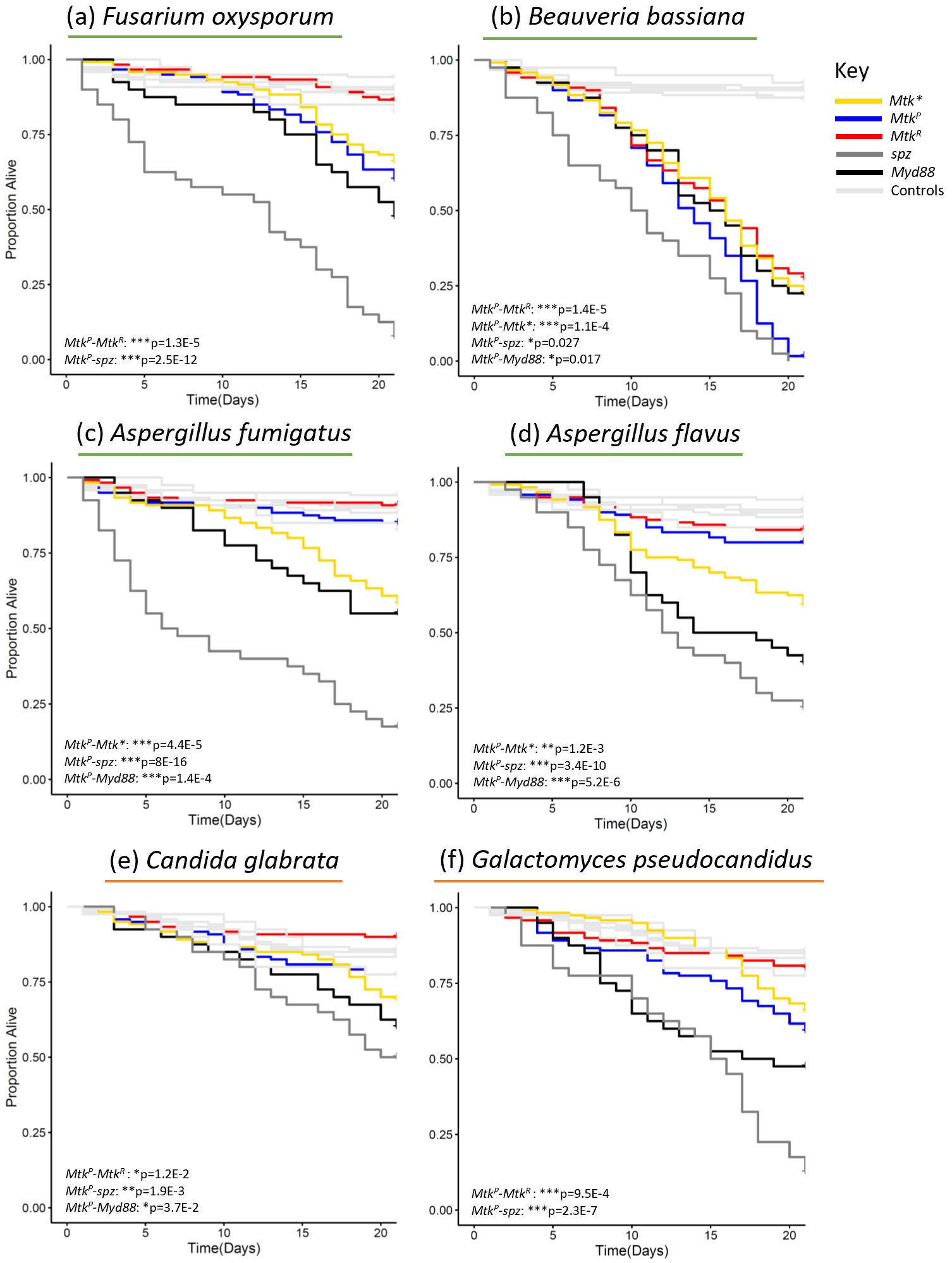

Supplement: S4 Fig — Infections were performed with the indicated microbes, using either spores (green underline) or yeast cultures (orange underline). Each line represents the survival of 120 flies (Mtk alleles and controls) or 40 flies (spz and Myd88) over a 21-day period for the same data graphed in Fig 2. Statistics based on Cox proportional hazard model (S2 Table). The experiment was performed twice, with combined results represented here. (TIF) [file pgen.1011155.s004.tif]

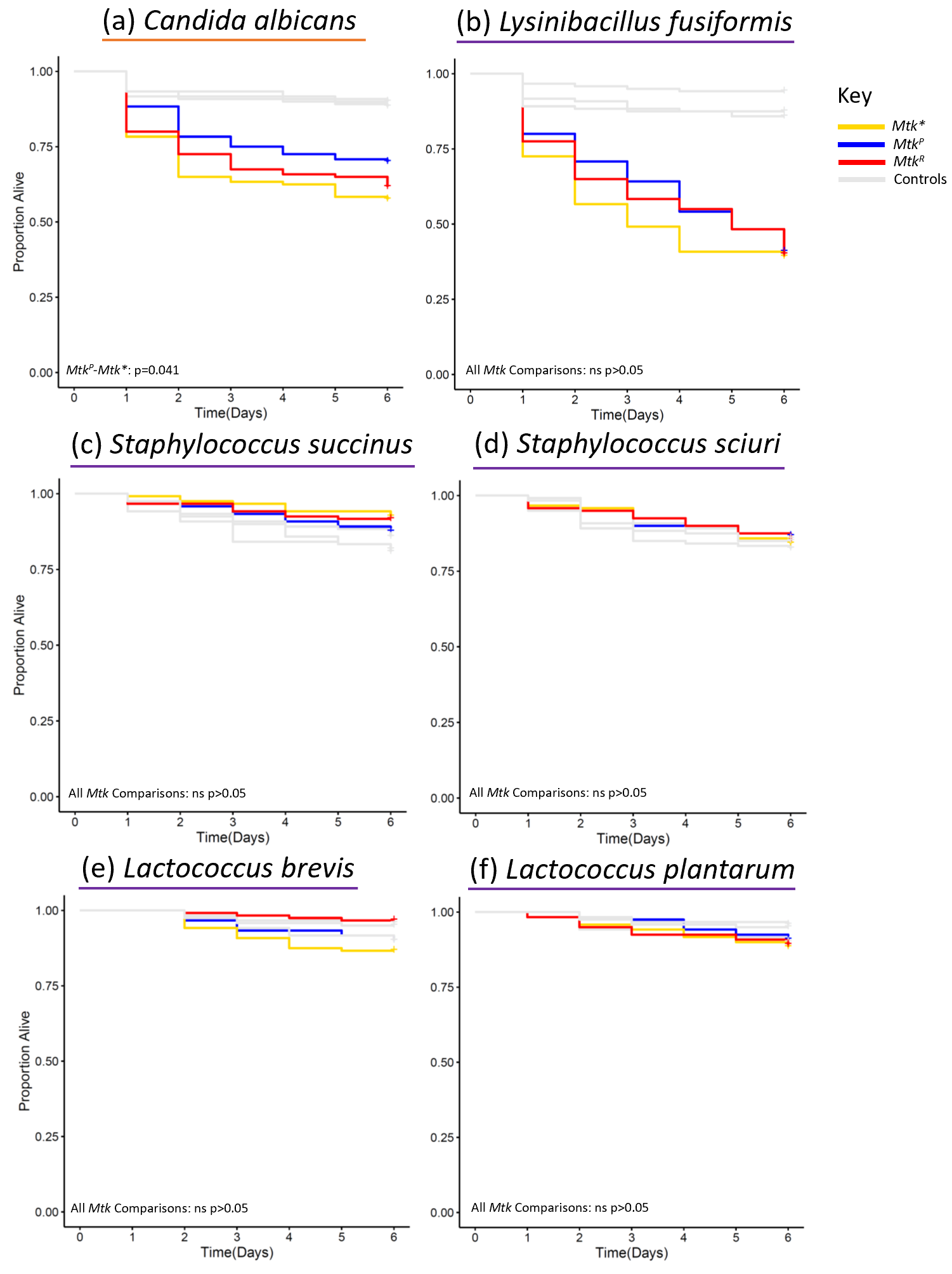

Supplement: S5 Fig — Infections were performed with the indicated microbes, using bacteria (purple underline) or yeast (orange underline). Each line represents the survival of 120 flies (Mtk alleles and controls) or 40 flies (spz and Myd88) over a 7-day period. Statistics based on Cox proportional hazard model (S2 Table). The experiment was performed twice, with combined results represented here. (TIF) [file pgen.1011155.s005.tif]

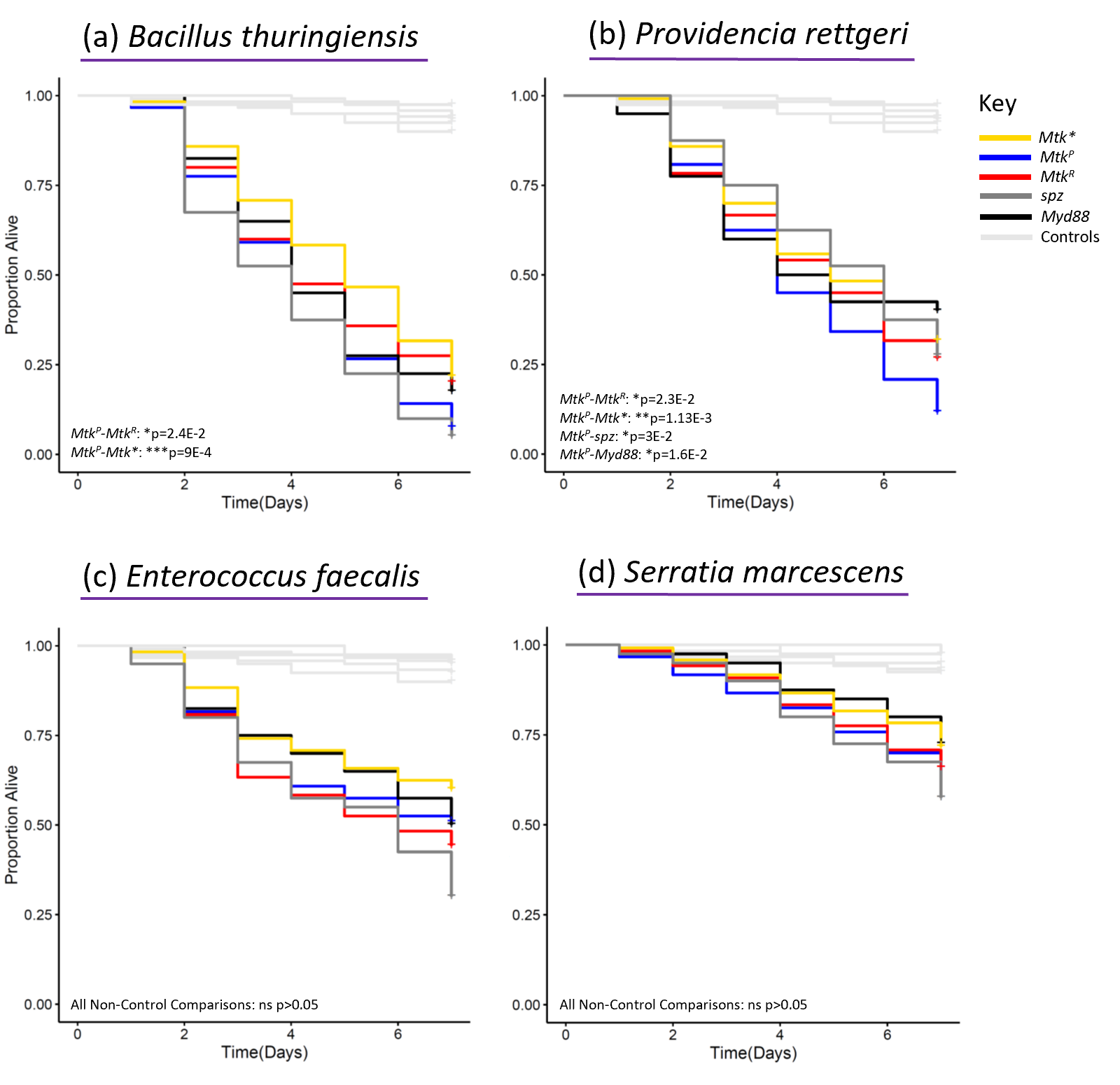

Supplement: S6 Fig — Infections were performed with the indicated microbes, using bacteria (purple underline). Each line represents the survival of 120 flies (Mtk alleles and controls) or 40 flies (spz and Myd88) over a 7-day period for the same data graphed in Fig 3. Statistics based on Cox proportional hazard model (S2 Table). The experiment was performed twice, with combined results represented here. (TIF) [file pgen.1011155.s006.tif]

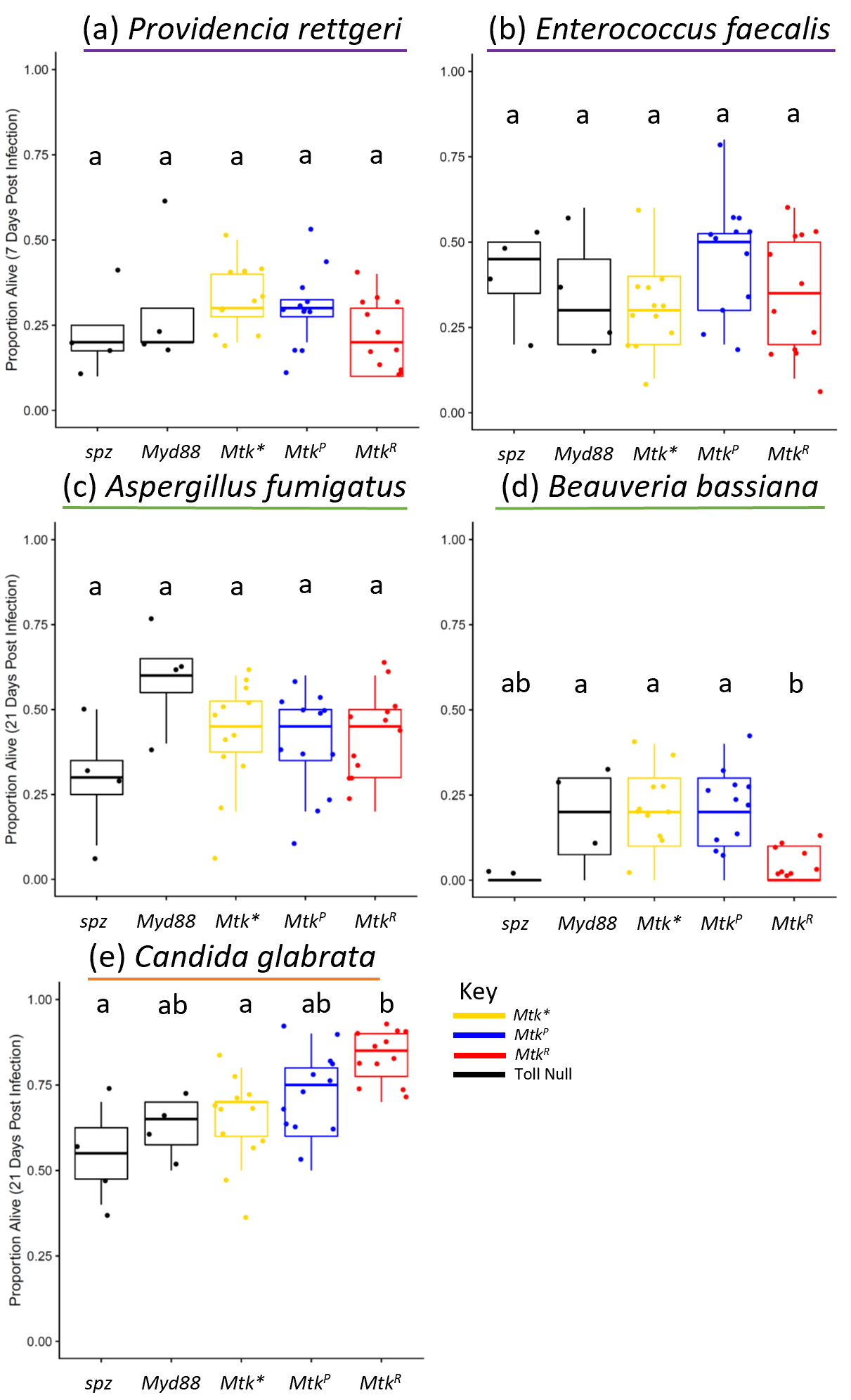

Supplement: S7 Fig — Infections were performed in females with the indicated microbes, using bacteria (purple underline), fungal spores (green underline), or yeast (orange underline). Each dot represents survival 21 days after infection for a vial starting with 10 females. Each set of data represents two independent experiments combined. Corresponding survival curves and controls for this experiment are shown in S8 Fig. The boxes indicate the interquartile range. Outer edges of the box indicate 25th (lower) and 75th (upper) percentiles and the middle line indicates 50th percentile (median). Whiskers represent maximum and minimum ranges of data within 1.5 times the interquartile range of the box. Letters indicate statistical significance groups, based on a logistic regression and Tukey post hoc test (S2 Table). (TIF) [file pgen.1011155.s007.tif]

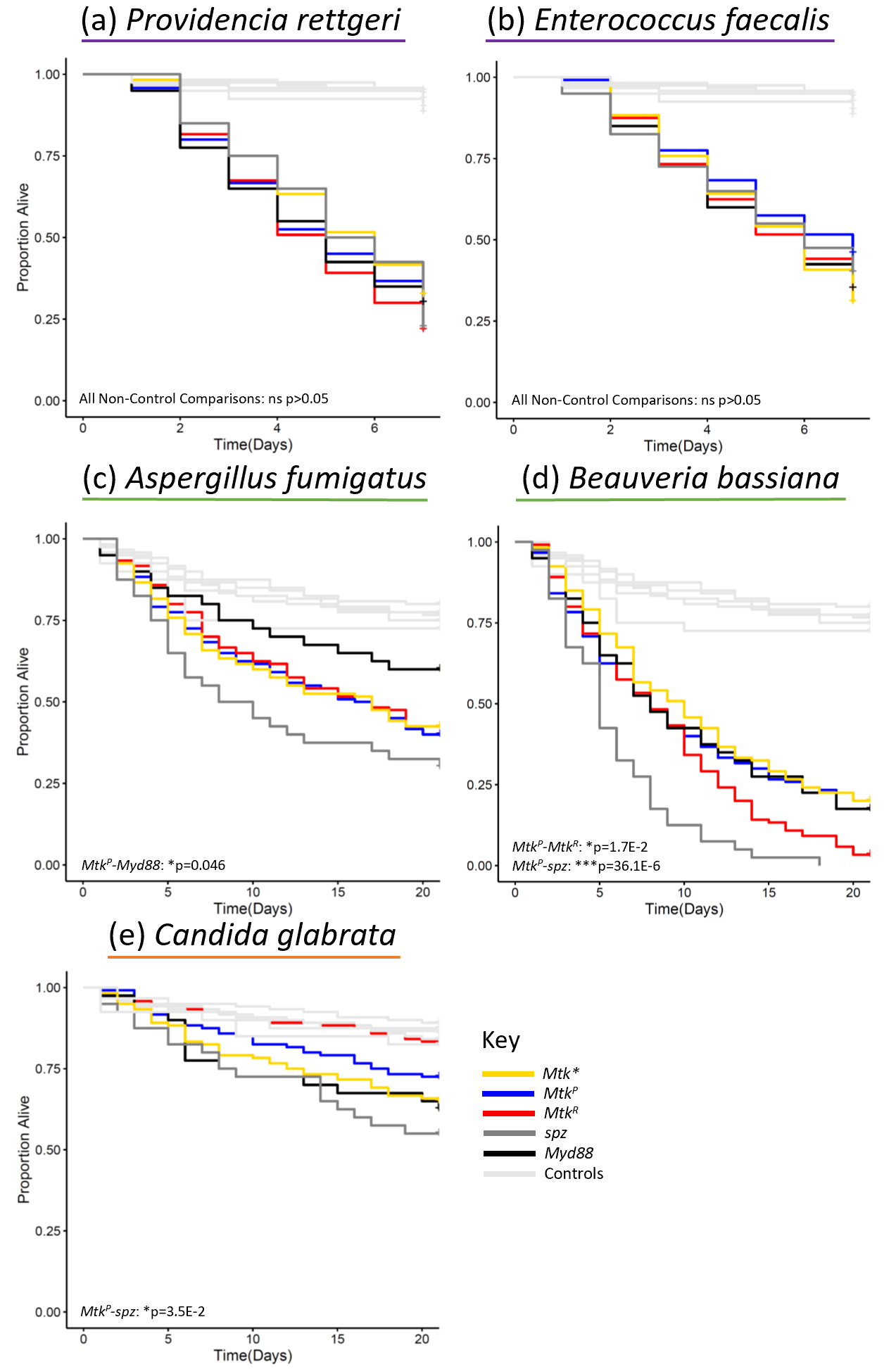

Supplement: S8 Fig — Infections were performed with the indicated microbes, using bacteria (purple underline), fungal spores (green underline), or yeast (orange underline). Each line represents the survival of 120 flies (Mtk alleles and controls) or 40 flies (spz and Myd88) over a 21-day period. Statistics based on Cox proportional hazard model (S2 Table). The experiment was performed twice, with combined results represented here. (TIF) [file pgen.1011155.s008.tif]

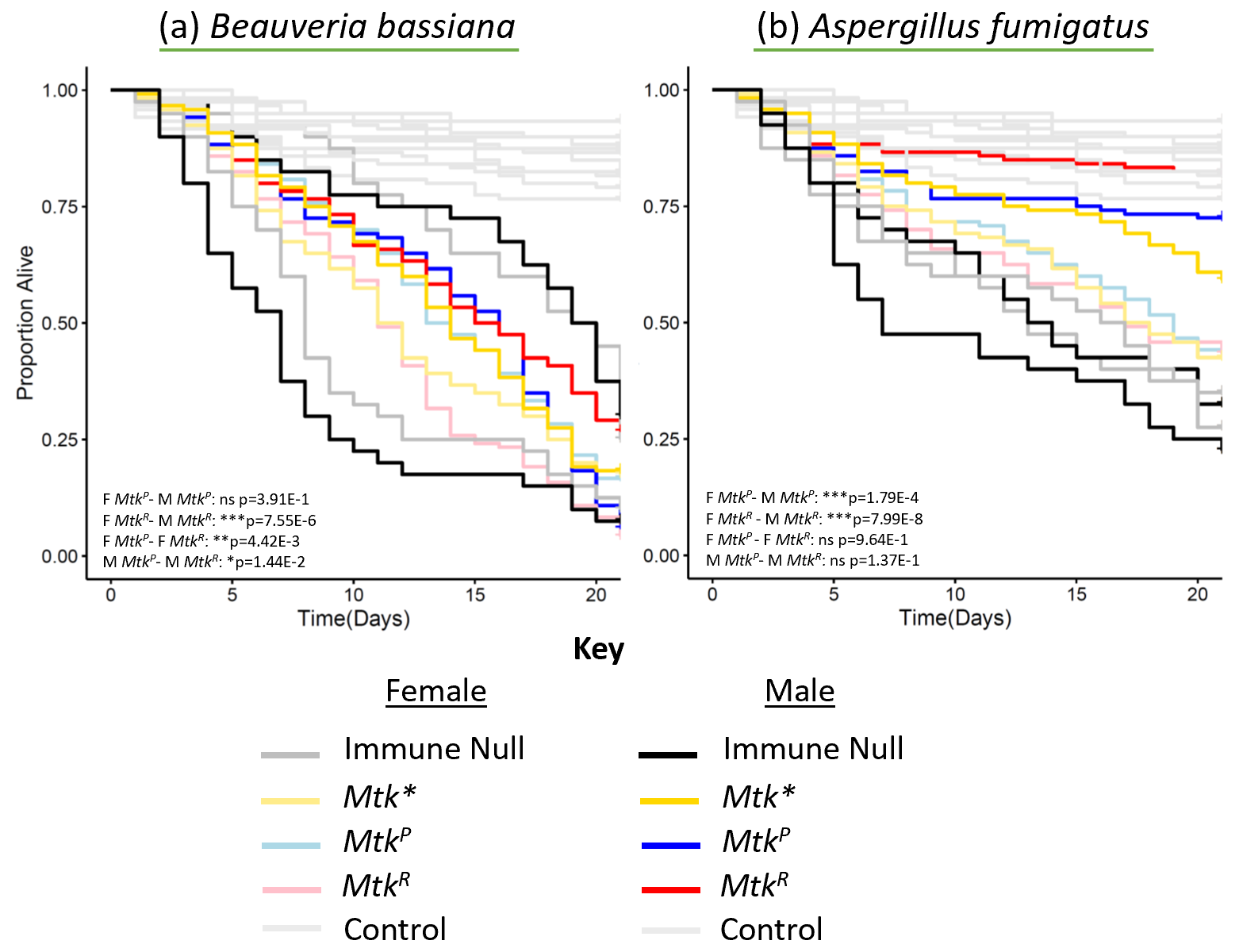

Supplement: S9 Fig — Infections were performed with the indicated microbes, using fungal spores (green underline). Each line represents the survival of 120 flies (Mtk alleles and controls) or 40 flies (spz and Myd88) over a 21-day period. Statistics based on Cox proportional hazard model, with false discovery rate corrections for a subset of contrasts (S2 Table). The experiment was performed twice, with combined results represented here. (TIF) [file pgen.1011155.s009.tif]

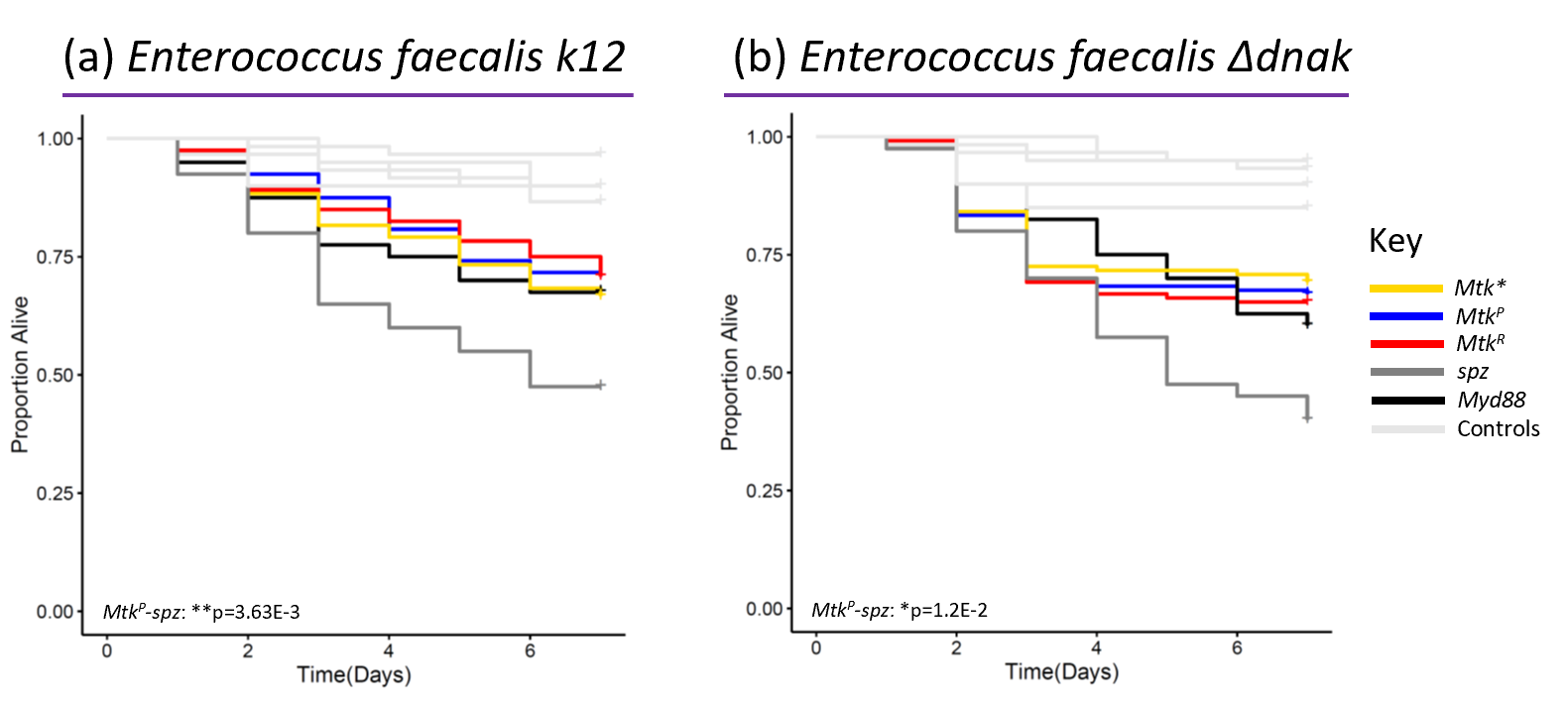

Supplement: S10 Fig — Infections were performed with E. faecalis (purple underline). Each line represents the survival of 120 flies (Mtk alleles and controls) or 40 flies (spz and Myd88) over a 21-day period. Statistics based on Cox proportional hazard model (S2 Table). The experiment was performed twice, with combined results represented here. (TIF) [file pgen.1011155.s010.tif]

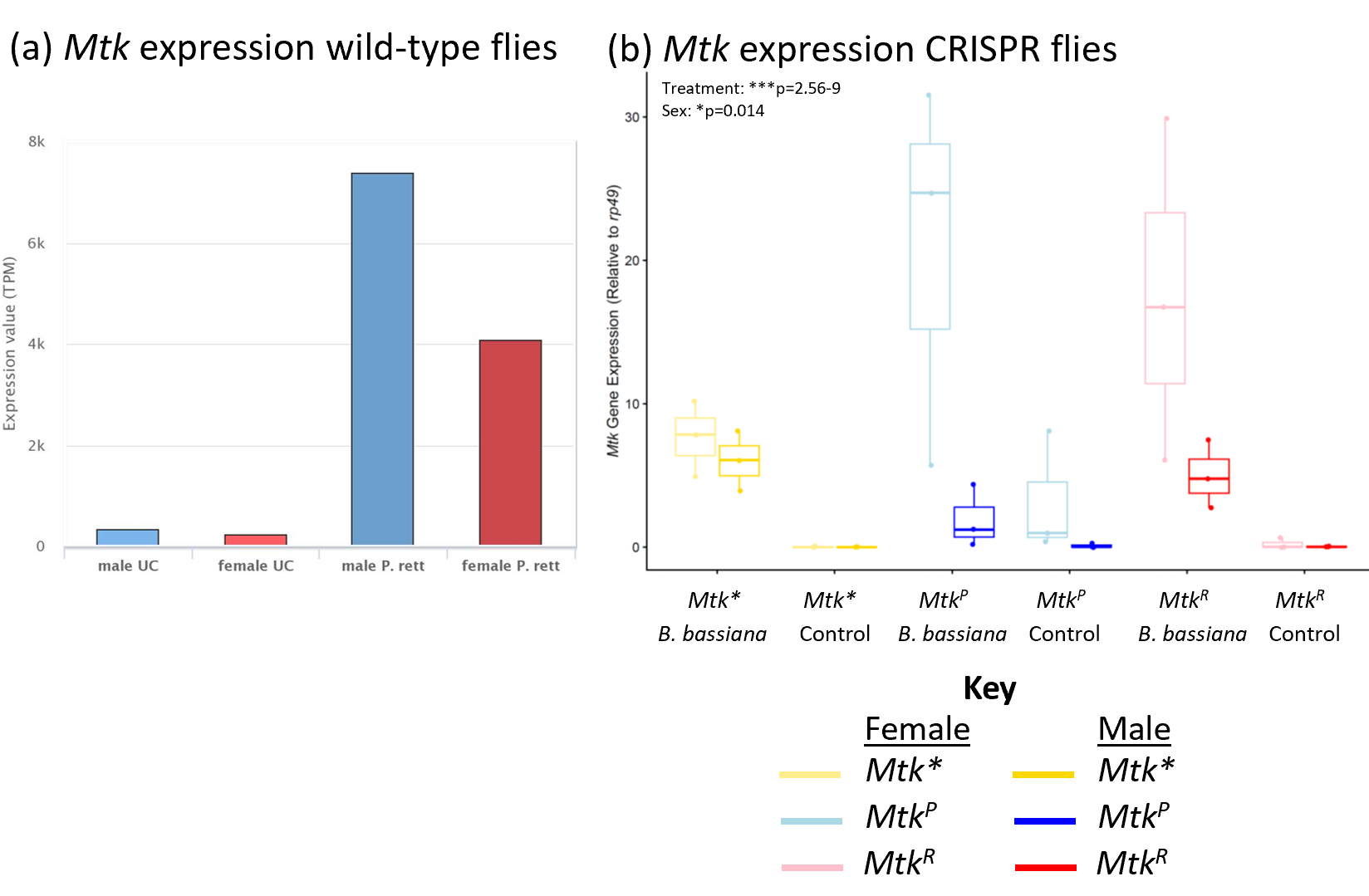

Supplement: S11 Fig — (a) Graph of Mtk expression levels (TPM, transcripts per million reads) in male and female Canton S flies either unchallenged with infection (UC) or challenged with Providencia rettgeri, 8 hours post-infection. Data from FlySexsick-seq database71. (b) qPCR data of Mtk gene expression with or without infection across CRISPR lines used in this study. Each data point represents a pool of 3 flies (whole bodies) either infected with Beauveria bassiana or not infected (control). qPCR based on levels of Mtk gene expression vs host rp49 housekeeping gene expression. Values denote 2-(Δct). Statistics are based on logistic regression and Tukey post-hoc test (S2 Table). (TIF) [file pgen.1011155.s011.tif]
