# Supplementary material for: A single amino acid polymorphism in natural Metchnikowin alleles of Drosophila results in systemic immunity and life history tradeoffs
Source: PLoS Genet. 2024 Mar 11;20(3):e1011155. doi: 10.1371/journal.pgen.1011155 (PMC10957085; doi:10.1371/journal.pgen.1011155)
Supplement: S3 Table — (DOCX) [file pgen.1011155.s014.docx]

|  | *Mtk**17b 369.1 | *Mtk**1bp 153.1 | *Mtk**6bp 306.2 | *Mtk^R^* 152.1 | *Mtk^R^* 369.1 | *Mtk^R^* 369.3 | *Mtk^P^* 305.1 | *Mtk^P^* 369.1 | *Mtk^P^* 373.1 |
| --- | --- | --- | --- | --- | --- | --- | --- | --- | --- |
| *Mtk^P^* 373.1 | 282 | 289 | 249 | 271 | 292 | 265 | 819 | 781 | - |
| *Mtk^P^* 369.1 | 637 | 632 | 578 | 574 | 631 | 610 | 1260 | - |  |
| *Mtk^P^* 305.1 | 669 | 679 | 617 | 678 | 701 | 667 | - |  |  |
| *Mtk^R^* 369.3 | 165 | 153 | 115 | 111 | 144 | - |  |  |  |
| *Mtk^R^* 369.1 | 199 | 177 | 145 | 140 | - |  |  |  |  |
| *Mtk^R^* 152.1 | 160 | 160 | 117 | - |  |  |  |  |  |
| *Mtk** 6bp 306.2 | 146 | 118 | - |  |  |  |  |  |  |
| *Mtk** 1bp 153.1 | 174 | - |  |  |  |  |  |  |  |
| *Mtk** 17bp 369.1 | - |  |  |  |  |  |  |  |  |
