# Supplementary material for: A single amino acid polymorphism in natural Metchnikowin alleles of Drosophila results in systemic immunity and life history tradeoffs
Source: PLoS Genet. 2024 Mar 11;20(3):e1011155. doi: 10.1371/journal.pgen.1011155 (PMC10957085; doi:10.1371/journal.pgen.1011155)
Supplement: S4 Table — (DOCX) [file pgen.1011155.s015.docx]

| **Name** | **Sequence** | **Notes** |  |
| --- | --- | --- | --- |
| Mtk_gRNA_target6 | 5’-AATCCTAACCAACCAAGACC-3’ | Guide RNA for Cas9 editing of *Mtk* sequence |  |
| Mtk_gRNA_target14 | 5’-CAATCCTAACCAACCAAGAC-3’ | Guide RNA for Cas9 editing of *Mtk* sequence |  |
| Mtk_ssDNA1 | 5'-TCAATGTGTTAACGACATCAGCAGTGTGAATTTCCCACT GTCAATTTAATAAATTCGACCTGGCCTTGGTTGGTTAGGAT TGAAGGGCGACGGCCTCGTATCGAAAAT GGGTCCCTGGTG-3’ | Single stranded donor for *Mtk* CRISPR/Cas9 editing |  |
| Mtk_F1 | 5’- ATTCCCGCCACCGAGCTAAG-3’ | Forward Primer |  |
| Mtk_R1 | 5’-TCAGCAGTGTGAATTTCCCACT-3’ | Reverse Primer |  |
| MtkPP4078F (qPCR) | 5’-ATGCAACTTAATCTTGGAGCGA-3’ | For qPCR, from FlyPrimerBank^80^ | |
| MtkPP4078F (qPCR) | 5’-GACGGCCTCGTATCGAAAATG-3’ | For qPCR, from FlyPrimerBank^80^ | |
| Rp49F | 5’-CGGTTACGGATCGAACAAGC-3’ | For qPCR | |
| Rp49R | 5’-CTTGCGCTTCTTGGAGGAGA-3’ | For qPCR | |
