# Supplementary material for: A single amino acid polymorphism in natural Metchnikowin alleles of Drosophila results in systemic immunity and life history tradeoffs
Source: PLoS Genet. 2024 Mar 11;20(3):e1011155. doi: 10.1371/journal.pgen.1011155 (PMC10957085; doi:10.1371/journal.pgen.1011155)
Supplement: S5 Table — (DOCX) [file pgen.1011155.s016.docx]

| **Species (strain)** | **Microbial Classification** | **Isolation Source** | **Stock Number or Isolated/Gifted By** |
| --- | --- | --- | --- |
| *Providencia rettgeri* (Dmel) | Gram negative | Wild *D. melanogaster* | Isolated by B. Lazzaro, State College, PA |
| *Bacillus thuringiensis* (Berliner) | Gram positive | *Ephestia kuehniella* | ATCC 10792 |
| *Enterococcus faecalis* (Dmel) | Gram positive | *D. melanogaster* hemolymph | Isolated by R. Unckless |
| *Serratia marcescens* | Gram negative | *D. melanogaster* hemolymph | Isolated by R. Unckless |
| *Lysinibacillus fusiformis* (Juneji) | Gram positive | *D. melanogaster* | Gift from B. Lazzaro |
| *Staphylococcus succinus* | Gram positive | *Drosophila* | Isolated by R. Unckless |
| *Staphylococcus sciuri* | Gram positive | *Drosophila* | Isolated by R. Unckless |
| *Lactococcus brevis* | Gram positive | *Drosophila* | Isolated by S. Mullinax |
| *Lactococcus plantarum* | Gram positive | *Drosophila* | Isolated by S. Mullinax |
| *Enterococcus faecalis* (K-12) | Gram positive | Feces | CGSC 7636 |
| *Enterococcus faecalis* (K-12 ΔdnaK) | Gram positive | Feces | CGSC 8342, derivative of CGSC 7636 |
| *Candida glabrata* (CBS 138) | Yeast | Feces | ATCC 2001 |
| *Candida auris* | Yeast | Clinical isolate | CDC B11903 |
| *Candida albicans* | Yeast | Clinical isolate | SC 5314 |
| *Galactomyces pseudocandidus* | Yeast | *Drosophila* | Isolated by I. Nevarez-Saenz |
| *Fusarium oxysporum* (f. sp. Lycopersici) | Filamentous fungus | Tomato | FGSC 9935 |
| *Beauveria bassiana* (GHA) | Filamentous fungus | *Locusta migratoria* | Gift from P. Shahrestani |
| *Aspergillus fumigatus* | Filamentous fungus | Clinical isolate | FGSC 1100 |
| *Aspergillus flavus* (NRRL 3357) | Filamentous fungus | Peanut | FGSC A1446 |
